# The ratio and difference of urine protein-to-creatinine ratio and albumin-to-creatinine ratio facilitate risk prediction of all-cause mortality

**DOI:** 10.1038/s41598-021-86541-3

**Published:** 2021-04-12

**Authors:** David Ray Chang, Hung-Chieh Yeh, I-Wen Ting, Chen-Yuan Lin, Han-Chun Huang, Hsiu-Yin Chiang, Shih-Ni Chang, Hsiu-Chen Tsai, Yen-Chun Lo, Chiung-Tzu Hsiao, Pei-Lun Chu, Chin-Chi Kuo

**Affiliations:** 1 Division of Nephrology, Department of Internal Medicine, China Medical University Hospital and College of Medicine, China Medical University, Taichung, Taiwan; 2 Division of Hematology and Oncology, Department of Internal Medicine, China Medical University Hospital and College of Medicine, China Medical University, Taichung, Taiwan; 3 Big Data Center, China Medical University Hospital and College of Medicine, China Medical University, 2, Yude Rd., North Dist., Taichung City, 404 Taiwan; 4 Department of Laboratory Medicine, China Medical University Hospital, Taichung, Taiwan; 5 Department of Medical Laboratory Science and Biotechnology, China Medical University, Taichung, Taiwan; 6 Division of Nephrology, Department of Internal Medicine, Fu-Jen Catholic University Hospital and College of Medicine, School of Medicine, Fu-Jen Catholic University, New Taipei City, Taiwan

**Keywords:** Chronic kidney disease, Glomerular diseases, Interstitial disease

## Abstract

The role of the difference and ratio of albuminuria (urine albumin-to-creatinine ratio, uACR) and proteinuria (urine protein-to-creatinine ratio, uPCR) has not been systematically evaluated with all-cause mortality. We retrospectively analyzed 2904 patients with concurrently measured uACR and uPCR from the same urine specimen in a tertiary hospital in Taiwan. The urinary albumin-to-protein ratio (uAPR) was derived by dividing uACR by uPCR, whereas urinary non-albumin protein (uNAP) was calculated by subtracting uACR from uPCR. Conventional severity categories of uACR and uPCR were also used to establish a concordance matrix and develop a corresponding risk matrix. The median age at enrollment was 58.6 years (interquartile range 45.4–70.8). During the 12,391 person-years of follow-up, 657 deaths occurred. For each doubling increase in uPCR, uACR, and uNAP, the adjusted hazard ratios (aHRs) of all-cause mortality were 1.29 (95% confidence interval [CI] 1.24–1.35), 1.12 (1.09–1.16), and 1.41 (1.34–1.49), respectively. For each 10% increase in uAPR, it was 1.02 (95% CI 0.98–1.06). The linear dose–response association with all-cause mortality was only observed with uPCR and uNAP. The 3 × 3 risk matrices revealed that patients with severe proteinuria and normal albuminuria had the highest risk of all-cause mortality (aHR 5.25, 95% CI 1.88, 14.63). uNAP significantly improved the discriminative performance compared to that of uPCR (c statistics: 0.834 vs. 0.828, p-value = 0.032). Our study findings advocate for simultaneous measurements of uPCR and uACR in daily practice to derive uAPR and uNAP, which can provide a better mortality prognostic assessment.

## Introduction

Proteinuria is a conventional therapeutic target, particularly in patients with chronic kidney disease (CKD) because proteinuria or albuminuria is associated with an increased risk of progression to end-stage renal disease (ESRD) and all-cause mortality^1,2^. In clinical practice, proteinuria is commonly quantified in random single-voided urine specimens and reported as a urine protein-to-creatinine ratio (uPCR) or an albumin-to-creatinine ratio (uACR)^3^. CKD guidelines recommend using uACR as the biomarker of choice to quantify proteinuria because its sensitivity is higher than that of uPCR for low levels of proteinuria. Professional care standards of hypertension and diabetes also specifically endorse the use of uACR to screen the development of hypertensive or diabetic nephropathy^4,5^. However, uPCR remains a rational alternative because its prognostic value was recently determined to be equivalent to uACR in patients with CKD^6^. Nevertheless, despite the continued controversy regarding universal screening of proteinuria in the general population, the preferable urinary biomarker for screening—uACR or uPCR—remains undetermined^7^.

The clinical significance of differences between uACR and uPCR is beyond the controversies surrounding the sensitivity issue. From the mechanistic viewpoint, albumin loss is more likely to be associated with injuries of the glomerular filtration apparatus, whereas protein loss (albumin and other proteins) represent a broader spectrum of kidney injuries, such as tubulointerstitial injury or systemic diseases such as multiple myeloma^7,8^. For instance, the absence of albuminuria (e.g., a negative urine dipstick for protein) but the presence of significant proteinuria indicates the possibility of non-albumin proteinuria (uNAP) including paraproteinuria^9^. The existing literature lacks a systematic evaluation of the clinical importance of uNAP despite the estimated prevalence of NAP in random urine samples of up to 10.1%^10^. Several studies have shown that uNAP may indicate higher nephrotoxicity and can offer insights into tubulointerstitial inflammation in lupus nephritis^11–13^. Although mathematical quantification of uNAP had been proposed by Hofmann et al. in 1995^14^, its first application in risk classification based on the urine albumin-to-protein proportion began 10 years later in a trial of children with kidney disease^15,16^. Ohisa et al. then determined that the urine albumin-to-protein ratio (uAPR) was useful in detecting glomerular disease in adult patients with hematuria^17^. Smith et al. further proved that uAPR correlated well with pathological tubulointerstitial damage and suggested a cut-off level of 0.4 for uAPR to discriminate tubulointerstitial and non-tubulointerstitial proteinuria^18^. However, the prognostic role of uNAP and uAPR in predicting all-cause mortality has not been clearly defined. Therefore, to fill this knowledge gap, we conducted a retrospective study using the large hospital-based database consisting of 2.7 million patients. We aimed to characterize clinical features and adverse outcomes of patients with different levels of uNAP and uAPR. Furthermore, we developed a classification matrix based on the severity grades of both uACR and uPCR and evaluated the prognostic performance of each cell in this classification matrix based on the mortality information from the National Mortality Registry (NMR).

## Results

### Distribution of uACR, uPCR, uAPR, and uNAP in the concordance matrix

Of the 2904 patients, the median age at enrollment was 58.6 years (IQR 45.4–70.8) with the median follow-up time of 3.32 years (IQR 1.41–6.28). Most patients had concordant proteinuria quantified using both uACR and uPCR (n = 2383, 82.1%), whereas 363 (12.5%) and 158 (5.4%) patients had non-albumin-predominant and albumin-predominant proteinuria, respectively (Supplementary Fig. S1 and Table 1).

**Table 1:** Baseline demographic and clinical characteristics stratified by the concordant pattern among categories of uPCR (< 150, ≧ 150 to < 500, and ≧ 500 mg/g creatinine) and uACR (< 30, ≧ 30 to < 300, and ≧ 300 mg/g creatinine).

Variable	Total (n = 2904)	Concordance proteinuria (n = 2383)	Non-albumin predominant proteinuria (n = 363)	Albumin-predominant proteinuria (n = 158)	P-value^†^
Age (year), median (IQR)	58.6 (45.4, 70.8)	57.7 (44.6, 69.9)	65.9 (55.0, 75.1)	58.3 (41.1, 69.3)	< 0.001
Female, n (%)	1207 (41.6)	987 (41.4)	148 (40.8)	72 (45.6)	0.6
Body mass index (kg/m^2^), median (IQR)	24.1 (21.8, 27.3)	24.4 (21.9, 27.4)	23.3 (21.1, 25.5)	23.8 (21.7, 28.3)	< 0.001
Follow-up duration (year), median (IQR)	3.32 (1.41, 6.28)	3.38 (1.53, 6.35)	2.36 (0.76, 5.09)	4.05 (2.52, 7.05)	< 0.001
**Baseline comorbidities, n (%)**					
CKD stage 1–2	1343 (47.3)	1133 (48.7)	115 (32.1)	95 (61.7)	< 0.001
CKD stage 3–5	1495 (52.7)	1193 (51.3)	243 (67.9)	59 (38.3)	
Diabetes	928 (32.1)	764 (32.2)	128 (35.3)	36 (23.1)	0.02
Hypertension	1245 (43.0)	1031 (43.4)	163 (44.9)	51 (32.7)	0.02
Cardiovascular disease	766 (26.5)	621 (26.2)	113 (31.1)	32 (20.5)	0.03
Cancer before enrollment	265 (9.1)	201 (8.4)	52 (14.3)	12 (7.6)	0.001
Cancer after enrollment	166 (5.7)	129 (5.4)	30 (8.3)	7 (4.4)	0.07
Cancer (before or after enrollment)	431 (14.8)	330 (13.9)	82 (22.6)	19 (12.0)	< 0.001
**Baseline medication profiles, n (%)**					
Pentoxifylline	389 (14.8)	325 (15.2)	48 (13.7)	16 (12.3)	0.5
Dipyridamole	91 (3.5)	74 (3.5)	13 (3.7)	4 (3.1)	0.9
Anti-platelet agents	683 (26.0)	555 (25.9)	104 (29.6)	24 (18.5)	0.04
NSAIDs	721 (27.5)	560 (26.1)	128 (36.5)	33 (25.4)	< 0.001
Contrast	479 (18.2)	351 (16.4)	112 (31.9)	16 (12.3)	< 0.001
**Antihypertension agents**					
ACEI	465 (17.7)	389 (18.1)	59 (16.8)	17 (13.1)	0.3
ARBs	919 (35.0)	769 (35.9)	102 (29.1)	48 (36.9)	0.04
Diuretics	1192 (45.4)	941 (43.9)	205 (58.4)	46 (35.4)	< 0.001
**Antidiabetic agents**					
OAD	724 (27.6)	600 (28.0)	93 (26.5)	31 (23.9)	0.5
Insulin	539 (20.5)	428 (20.0)	104 (29.6)	7 (5.4)	< 0.001
**Baseline biochemical profiles, median (IQR)**					
eGFR (mL/min/1.73m^2^)	56.8 (27.8, 91.8)	58.3 (28.4, 93.1)	39.3 (17.1, 71.3)	73.1 (51.5, 97.5)	< 0.001
Serum creatinine (mg/dL)	1.23 (0.86, 2.16)	1.21 (0.86, 2.12)	1.69 (1.01, 3.15)	1.00 (0.79, 1.32)	< 0.001
Blood urea nitrogen (mg/dL)	20.0 (13.0, 36.0)	19.0 (13.0, 35.0)	26.0 (17.0, 47.0)	15.0 (11.0, 21.0)	< 0.001
Serum uric acid (mg/dL)	6.50 (5.20, 8.00)	6.60 (5.30, 8.00)	6.60 (4.75, 8.50)	6.10 (5.20, 7.40)	0.05
Sodium (mmol/L)	138 (136, 140)	138 (136, 140)	137 (134, 140)	138 (137, 140)	0.001
Potassium (mmol/L)	4.10 (3.70, 4.50)	4.10 (3.80, 4.50)	3.90 (3.50, 4.40)	4.00 (3.80, 4.40)	< 0.001
Calcium (mg/dL)	8.70 (8.20, 9.20)	8.70 (8.20, 9.20)	8.60 (7.93, 9.10)	9.10 (8.80, 9.40)	< 0.001
Phosphate (mg/dL)	4.00 (3.40, 4.80)	4.00 (3.50, 4.80)	4.00 (3.10, 4.90)	3.60 (3.30, 4.00)	0.002
Serum albumin (g/dL)	4.00 (3.30, 4.40)	4.00 (3.40, 4.40)	3.65 (2.90, 4.20)	4.30 (4.00, 4.60)	< 0.001
Hemoglobin (g/dL)	11.9 (9.8, 13.8)	12.0 (9.9, 13.9)	10.8 (9.2, 12.4)	13.0 (11.5, 14.7)	< 0.001
Total cholesterol (mg/dL)	181 (152, 214)	185 (155, 217)	156 (134, 186)	181 (157, 213)	< 0.001
Triglyceride (mg/dL)	129 (85, 195)	131 (86, 196)	117 (81, 199)	121 (82, 182)	0.08
Glucose (mg/dL)	116 (99, 159)	116 (98, 157)	129 (102, 176)	109 (94, 131)	< 0.001
Urine creatinine (mg/dL)	92.3 (59.4, 144.0)	97.9 (61.8, 149.3)	65.3 (48.5, 96.5)	104.9 (71.5, 145.0)	< 0.001
uPCR (mg/g)	310 (101, 1,608)	308 (90, 1,990)	579 (247, 970)	125 (109, 137)	< 0.001
uACR (mg/g)	86 (13, 757)	108 (11, 1,152)	75 (19, 169)	46 (36, 55)	< 0.001
uAPR (%)	30.0 (10.8, 57.5)	36.1 (12.3, 60.9)	10.5 (5.0, 18.3)	38.0 (31.7, 46.4)	< 0.001
uNAP (mg/g)	186 (80, 683)	174 (77, 743)	470 (236, 843)	75 (63, 92)	< 0.001

*ACEI* angiotensin-converting-enzyme inhibitors, *ARBs* angiotensin receptor blockers, *CKD* chronic kidney disease, *eGFR* estimated glomerular filtration rate, *NSAIDs* nonsteroidal anti-inflammatory drugs, *OAD* oral antidiabetic agents, *uPCR* urine protein-to-creatinine ratio, *uACR* urine albumin-to-creatinine ratio, *uAPR* urine albumin-to-protein ratio, *uNAP* urine non-albumin proteinuria.

^†^P-values are calculated by Kruskal–Wallis test for continuous variables and Chi-square test for categorical variables.

**Figure 1:**
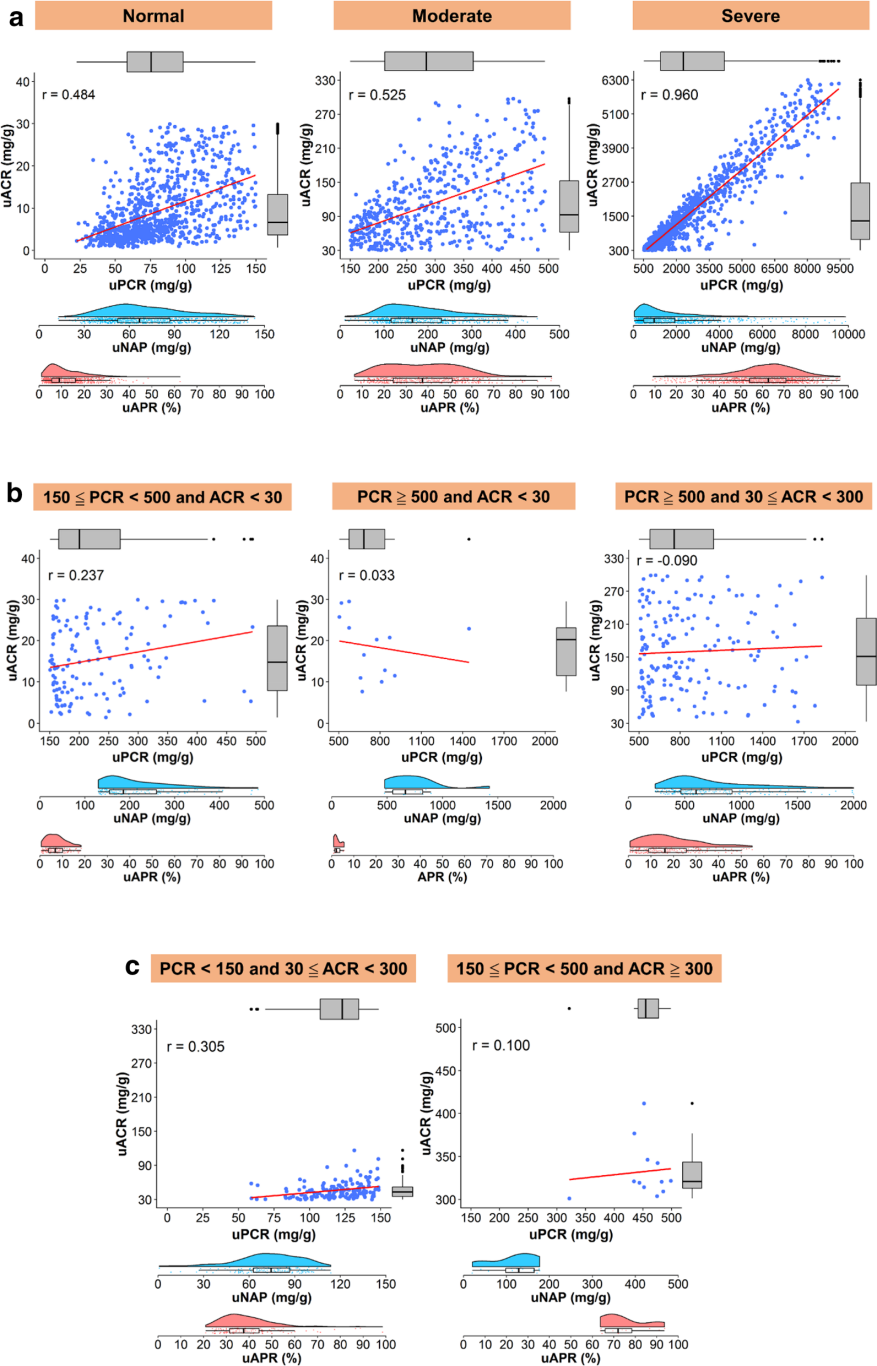
(**a**) The correlation between uPCR and uACR based on the severity grade of concordant proteinuria. The distribution of uAPR and uNAP is shown using a raincloud plot that combines a boxplot, raw jittered data, and the probability density curve. *r*, Pearson correlation. (**b**) The correlation between uPCR and uACR based on different categories of non-albumin predominant proteinuria. The distribution of uAPR and uNAP is shown using a raincloud plot that combines a boxplot, raw jittered data, and the probability density curve. *r*, Pearson correlation. (**c**) The correlation between uPCR and uACR by the albumin predominant proteinuria between uPCR and uACR. Numbers along the box plot refer to the median and interquartile range. The distribution of uAPR and uNAP are shown using a raincloud plot that combines a boxplot, raw jittered data, and the probability density curve. *r*, Pearson correlation.

In the concordant group, uAPR gradually increased from a median of 9.0% in patients without albuminuria and proteinuria to 62.7% in patients with severe albuminuria and concomitant proteinuria (Fig. 1a). The median (IQR) of uNAP was gradually increased from 66.8, 164.5, 975.5 across the severity categories of concordance groups (Fig. 1a).

In the non-albumin-predominant group, uAPR was low to a median of 2.0% in patients with severe proteinuria but without albuminuria. By contrast, uAPR was elevated to a median of 37.6% in patients with severe albuminuria but without proteinuria (Table 1 and Fig. 1b,c).

Compared with the concordant group, patients in the non-albumin-predominant group were older with lower BMI besides having a higher prevalence of diabetes, hypertension, CVD, and cancer, and a higher likelihood of exposure to nonsteroidal anti-inflammatory drugs (NSAIDs) or contrast (Table 1). Patients in the non-albumin-predominant group also had a higher prevalence of CKD 3–5. The use of ACEI was similar among the three groups; however, ARB was less frequently prescribed in the non-albumin-predominant group. Among the three groups, patients in the non-albumin-predominant group tended to have lower levels of eGFR, serum albumin, hemoglobin, total cholesterol, triglyceride, and urine creatinine levels (Table 1).

### Clinical characteristics based on the uAPR quartile

Only patients in the lowest quartile of uAPR were younger (median 53.7 years) than the overall median age (median 58.6 years). No sex differences were observed across the uAPR quartiles (Table 2). Patients in the highest quartile of uAPR had greater BMI, higher prevalence of advance CKD (Stage 3–5), diabetes, hypertension, and CVD with corresponding use of ACEI, ARB, diuretics, and anti-diabetic agents compared with those in the lowest uAPR quartile. By contrast, patients in the lowest quartile of uAPR had a higher likelihood of exposure to NSAID and radiocontrast. Regarding the biochemical parameters, serum creatinine, urea nitrogen, uric acid, potassium, phosphate, total cholesterol, triglyceride, uPCR, and uACR showed increasing trends across the increasing quartiles of uAPR. By contrast, decreasing trends were observed in levels of serum calcium, albumin, hemoglobin, and urine creatinine across the increasing quartiles of uAPR (Table 2).

**Table 2:** Baseline demographic and clinical characteristics stratified by quartiles of urine albumin-to-protein ratio (uAPR).

Variable	Total (n = 2904)	uAPR ≦ 10.8 (n = 723)	10.8 < uAPR ≦ 30.0 (n = 737)	30.0 < uAPR ≦ 57.5 (n = 720)	uAPR > 57.5 (n = 724)	P-value^†^	P for trend^‡^
Age (year), median (IQR)	58.6 (45.4, 70.8)	53.7 (40.2, 66.3)	59.8 (46.8, 72.5)	61.9 (49.7, 73.8)	58.1 (45.5, 69.7)	< 0.001	< 0.001
Female, n (%)	1207 (41.6)	275 (38.0)	318 (43.2)	302 (41.9)	312 (43.1)	0.2	0.09
Body mass index (kg/m^2^), median (IQR)	24.1 (21.8, 27.3)	23.5 (21.3, 25.8)	23.9 (21.3, 26.7)	24.0 (21.8, 27.4)	24.8 (22.5, 28.3)	< 0.001	< 0.001
Follow-up duration (year), median (IQR)	3.32 (1.41, 6.28)	3.33 (1.44, 5.73)	3.47 (1.45, 6.65)	3.17 (1.27, 6.46)	3.25 (1.54, 6.01)	0.6	0.9
**Baseline comorbidities, n (%)**							
CKD stage 1–2	1343 (47.3)	485 (69.8)	367 (51.4)	256 (36.0)	235 (32.7)	< 0.001	< 0.001
CKD stage 3–5	1495 (52.7)	210 (30.2)	347 (48.6)	455 (64.0)	483 (67.3)		
Diabetes	928 (32.1)	144 (19.9)	226 (30.9)	269 (37.4)	289 (40.1)	< 0.001	< 0.001
Hypertension	1245 (43.0)	167 (23.1)	306 (41.9)	373 (51.8)	399 (55.3)	< 0.001	< 0.001
Cardiovascular disease	766 (26.5)	132 (18.3)	198 (27.1)	221 (30.8)	215 (29.8)	< 0.001	< 0.001
Cancer before enrollment	265 (9.1)	61 (8.4)	80 (10.9)	64 (8.9)	60 (8.3)	0.3	0.6
Cancer after enrollment	166 (5.7)	39 (5.4)	48 (6.5)	50 (6.9)	29 (4.0)	0.07	0.3
Cancer (before or after enrollment)	431 (14.8)	100 (13.8)	128 (17.4)	114 (15.8)	89 (12.3)	0.04	0.3
**Baseline medication profiles, n (%)**							
Pentoxifylline	389 (14.8)	40 (6.6)	72 (10.7)	120 (17.9)	157 (23.3)	< 0.001	< 0.001
Dipyridamole	91 (3.5)	13 (2.1)	21 (3.1)	28 (4.2)	29 (4.3)	0.1	0.02
Anti-platelet agents	683 (26.0)	129 (21.2)	171 (25.3)	196 (29.3)	187 (27.8)	0.006	0.003
NSAIDs	721 (27.5)	190 (31.2)	212 (31.4)	176 (26.3)	143 (21.3)	< 0.001	< 0.001
Contrast	479 (18.2)	142 (23.3)	146 (21.6)	108 (16.1)	83 (12.3)	< 0.001	< 0.001
**Antihypertension agents**							
ACEI	465 (17.7)	50 (8.2)	107 (15.9)	160 (23.9)	148 (22.0)	< 0.001	< 0.001
ARBs	919 (35.0)	108 (17.7)	217 (32.2)	267 (39.9)	327 (48.6)	< 0.001	< 0.001
Diuretics	1192 (45.4)	217 (35.6)	289 (42.8)	326 (48.7)	360 (53.5)	< 0.001	< 0.001
**Antidiabetic agents**							
OAD	724 (27.6)	116 (19.1)	174 (25.8)	204 (30.5)	230 (34.2)	< 0.001	< 0.001
Insulin	539 (20.5)	81 (13.3)	131 (19.4)	169 (25.3)	158 (23.5)	< 0.001	< 0.001
**Baseline biochemical profiles, median (IQR)**							
eGFR (mL/min/1.73m^2^)	56.8 (27.8, 91.8)	84.0 (53.0, 104.6)	62.0 (31.9, 94.0)	46.4 (19.7, 76.3)	40.9 (19.5, 71.7)	< 0.001	< 0.001
Serum creatinine (mg/dL)	1.23 (0.86, 2.16)	0.96 (0.77, 1.33)	1.16 (0.81, 1.88)	1.45 (1.00, 2.87)	1.59 (1.03, 2.91)	< 0.001	< 0.001
Blood urea nitrogen (mg/dL)	20.0 (13.0, 36.0)	15.0 (11.0, 21.0)	18.0 (12.0, 31.5)	24.0 (15.0, 44.0)	26.0 (16.0, 45.0)	< 0.001	< 0.001
Serum uric acid (mg/dL)	6.50 (5.20, 8.00)	5.80 (4.80, 7.10)	6.25 (4.90, 7.80)	6.60 (5.50, 8.00)	7.20 (6.00, 8.60)	< 0.001	< 0.001
Sodium (mmol/L)	138 (136, 140)	138 (135, 140)	138 (136, 140)	138 (135, 140)	138 (136, 139)	0.7	0.3
Potassium (mmol/L)	4.10 (3.70, 4.50)	4.00 (3.70, 4.30)	4.00 (3.70, 4.40)	4.10 (3.70, 4.50)	4.30 (3.80, 4.60)	< 0.001	< 0.001
Calcium (mg/dL)	8.70 (8.20, 9.20)	8.90 (8.30, 9.30)	8.80 (8.23, 9.20)	8.70 (8.20, 9.20)	8.70 (8.10, 9.10)	0.007	0.001
Phosphate (mg/dL)	4.00 (3.40, 4.80)	3.70 (3.05, 4.50)	3.70 (3.20, 4.50)	4.10 (3.60, 4.90)	4.30 (3.70, 5.10)	< 0.001	< 0.001
Serum albumin (g/dL)	4.00 (3.30, 4.40)	4.10 (3.50, 4.50)	4.10 (3.60, 4.40)	3.90 (3.20, 4.30)	3.70 (3.10, 4.10)	< 0.001	< 0.001
Hemoglobin (g/dL)	11.9 (9.8, 13.8)	12.5 (10.6, 14.1)	12.0 (10.1, 13.8)	10.9 (9.3, 13.3)	11.9 (9.8, 13.9)	< 0.001	< 0.001
Total cholesterol (mg/dL)	181 (152, 214)	176 (146, 210)	170 (144, 201)	179 (153, 207)	197 (165, 238)	< 0.001	< 0.001
Triglyceride (mg/dL)	129 (85, 195)	113 (74, 173)	124 (82, 178)	127 (85, 184)	152 (103, 231)	< 0.001	< 0.001
Glucose (mg/dL)	116 (99, 159)	112 (97, 154)	116 (99, 157)	119 (100, 168)	119 (98, 160)	0.05	0.05
Urine creatinine (mg/dL)	92.3 (59.4, 144.0)	121.3 (74.3, 169.8)	98.2 (63.3, 151.4)	83.8 (54.4, 127.6)	78.0 (53.0, 115.9)	< 0.001	< 0.001
uPCR (mg/g)	310 (101, 1,608)	89 (62, 162)	138 (87, 380)	458 (185, 1,733)	2416 (1,008, 5072)	< 0.001	< 0.001
uACR (mg/g)	86 (13, 757)	5 (3, 10)	28 (15, 76)	203 (83, 832)	1682 (697, 3474)	< 0.001	< 0.001
uAPR (%)	30.0 (10.8, 57.5)	6.0 (4.0, 8.2)	18.3 (14.4, 23.8)	45.7 (37.7, 52.6)	67.8 (62.9, 74.7)	< 0.001	< 0.001
uNAP (mg/g)	186 (80, 683)	83 (58, 156)	112 (69, 304)	249 (105, 931)	674 (280, 1556)	< 0.001	< 0.001

*ACEI* angiotensin-converting-enzyme inhibitors, *ARBs* angiotensin receptor blockers, *CKD* chronic kidney disease, *eGFR* estimated glomerular filtration rate, *NSAIDs* nonsteroidal anti-inflammatory drugs, *OAD* oral antidiabetic agents, *uPCR* urine protein-to-creatinine ratio, *uACR* urine albumin-to-creatinine ratio, *uAPR* urine albumin-to-protein ratio, *uNAP* urine non-albumin proteinuria.

^†^P-values are calculated by Kruskal–Wallis test for continuous variables and Chi-square test for categorical variables.

^‡^P-values for trend are calculated by Spearman's correlation for continuous variables and by Cochran-Armitage trend test for binary variables.

### Clinical characteristics based on the uNAP quartile

Chronic diseases including CKD, diabetes, hypertension, and CVD were more prevalent among patients in the highest quartile of uNAP (uNAP > 682.9 mg/g creatinine) than those in the lower quartiles (Table 3). A corresponding higher use of anti-hypertensive drugs and oral hypoglycemic medications was observed in patients in the highest quartile of uNAP (Table 3). Compared with patients in the lowest quartile of uNAP, those with uNAP above 80.3 mg/g creatinine tended to have a higher prevalence and incidence of cancer. Significant trends of increasing blood urea nitrogen, serum creatinine, uric acid, phosphorus, triglyceride, uPCR, uACR, and uAPR were noted across the increasing quartiles of uNAP. By contrast, decreasing trends of serum albumin and hemoglobin were observed across the increasing uNAP quartiles (Table 3). Baseline clinical characteristics based on quartiles of uACR and uPCR are also provided in Supplementary Tables S1 and S2, respectively.

**Table 3:** Baseline demographic and clinical characteristics stratified by quartiles of urine non-albumin proteinuria (uNAP) defined by subtracting uACR from uPCR ratio.

Variable	Total (n = 2904)	uNAP ≦ 80.3 (n = 725)	80.3 < uNAP ≦ 186.3 (n = 727)	186.3 < uNAP ≦ 682.9 (n = 726)	uNAP > 682.9 (n = 726)	P-value^†^	P for trend^‡^
Age (year), median (IQR)	58.6 (45.4, 70.8)	49.9 (36.6, 60.8)	59.1 (47.4, 71.9)	62.7 (51.7, 74.3)	62.2 (49.6, 73.8)	< 0.001	< 0.001
Female, n (%)	1207 (41.6)	269 (37.1)	310 (42.6)	288 (39.7)	340 (46.8)	0.001	0.001
Body mass index (kg/m^2^), median (IQR)	24.1 (21.8, 27.3)	24.3 (21.4, 27.1)	24.4 (22.1, 27.2)	24.0 (21.8, 27.3)	24.1 (21.8, 27.4)	0.8	0.7
Follow-up duration (year), median (IQR)	3.32 (1.41, 6.28)	3.39 (1.79, 6.21)	4.19 (2.06, 7.15)	3.30 (1.25, 6.08)	2.45 (0.88, 5.13)	< 0.001	< 0.001
**Baseline comorbidities, n (%)**							
CKD stage 1–2	1343 (47.3)	539 (78.1)	431 (60.8)	250 (34.9)	123 (17.0)	< 0.001	< 0.001
CKD stage 3–5	1495 (52.7)	151 (21.9)	278 (39.2)	466 (65.1)	600 (83.0)		
Diabetes	928 (32.1)	120 (16.7)	215 (29.7)	262 (36.2)	331 (45.6)	< 0.001	< 0.001
Hypertension	1245 (43.0)	181 (25.1)	282 (38.9)	364 (50.4)	418 (57.6)	< 0.001	< 0.001
Cardiovascular disease	766 (26.5)	107 (14.9)	173 (23.9)	218 (30.2)	268 (37.0)	< 0.001	< 0.001
Cancer before enrollment	265 (9.1)	40 (5.5)	70 (9.6)	87 (12.0)	68 (9.4)	< 0.001	0.004
Cancer after enrollment	166 (5.7)	21 (2.9)	50 (6.9)	48 (6.6)	47 (6.5)	0.002	0.007
Cancer (before or after enrollment)	431 (14.8)	61 (8.4)	120 (16.5)	135 (18.6)	115 (15.8)	< 0.001	< 0.001
**Baseline medication profiles, n (%)**							
Pentoxifylline	389 (14.8)	41 (7.1)	72 (11.1)	139 (20.0)	137 (19.5)	< 0.001	< 0.001
Dipyridamole	91 (3.5)	9 (1.6)	14 (2.2)	33 (4.8)	35 (5.0)	0.001	< 0.001
Anti-platelet agents	683 (26.0)	104 (18.0)	160 (24.6)	213 (30.7)	206 (29.3)	< 0.001	< 0.001
NSAIDs	721 (27.5)	154 (26.7)	176 (27.0)	205 (29.5)	186 (26.4)	0.5	0.9
Contrast	479 (18.2)	88 (15.3)	107 (16.4)	152 (21.9)	132 (18.8)	0.01	0.02
**Antihypertension agents**							
ACEI	465 (17.7)	50 (8.7)	100 (15.4)	123 (17.7)	192 (27.3)	< 0.001	< 0.001
ARBs	919 (35.0)	146 (25.3)	208 (32.0)	265 (38.2)	300 (42.6)	< 0.001	< 0.001
Diuretics	1192 (45.4)	141 (24.4)	220 (33.8)	327 (47.1)	504 (71.6)	< 0.001	< 0.001
**Antidiabetic agents**							
OAD	724 (27.6)	105 (18.2)	190 (29.2)	195 (28.1)	234 (33.2)	< 0.001	< 0.001
Insulin	539 (20.5)	33 (5.7)	85 (13.1)	178 (25.7)	243 (34.5)	< 0.001	< 0.001
**Baseline biochemical profiles, median (IQR)**							
eGFR (mL/min/1.73m^2^)	56.8 (27.8, 91.8)	89.3 (63.1, 107.0)	70.0 (47.3, 96.7)	44.4 (24.7, 75.6)	19.6 (8.4, 45.6)	< 0.001	< 0.001
Serum creatinine (mg/dL)	1.23 (0.86, 2.16)	0.92 (0.76, 1.18)	1.04 (0.79, 1.42)	1.48 (1.00, 2.44)	2.84 (1.45, 5.75)	< 0.001	< 0.001
Blood urea nitrogen (mg/dL)	20.0 (13.0, 36.0)	13.0 (10.0, 17.0)	17.0 (12.0, 22.0)	23.0 (15.0, 37.0)	42.0 (23.0, 68.0)	< 0.001	< 0.001
Serum uric acid (mg/dL)	6.50 (5.20, 8.00)	5.90 (4.80, 7.10)	6.10 (5.00, 7.40)	7.00 (5.50, 8.50)	7.30 (6.00, 8.90)	< 0.001	< 0.001
Sodium (mmol/L)	138 (136, 140)	138 (137, 140)	138 (136, 140)	137 (135, 139)	137 (135, 140)	< 0.001	< 0.001
Potassium (mmol/L)	4.10 (3.70, 4.50)	4.00 (3.80, 4.30)	4.00 (3.80, 4.40)	4.10 (3.70, 4.50)	4.20 (3.70, 4.60)	0.02	0.002
Calcium (mg/dL)	8.70 (8.20, 9.20)	9.10 (8.80, 9.40)	9.00 (8.60, 9.30)	8.90 (8.40, 9.20)	8.30 (7.80, 8.80)	< 0.001	< 0.001
Phosphate (mg/dL)	4.00 (3.40, 4.80)	3.60 (3.20, 4.10)	3.70 (3.10, 4.20)	3.90 (3.30, 4.50)	4.50 (3.70, 5.60)	< 0.001	< 0.001
Serum albumin (g/dL)	4.00 (3.30, 4.40)	4.40 (4.10, 4.60)	4.20 (3.90, 4.50)	3.90 (3.50, 4.30)	3.20 (2.60, 3.70)	< 0.001	< 0.001
Hemoglobin (g/dL)	11.9 (9.8, 13.8)	13.6 (12.1, 14.8)	13.2 (11.7, 14.5)	11.6 (10.0, 13.2)	9.9 (8.6, 11.7)	< 0.001	< 0.001
Total cholesterol (mg/dL)	181 (152, 214)	184 (159, 213)	179 (155, 210)	180 (149, 208)	182 (149, 229)	0.07	0.8
Triglyceride (mg/dL)	129 (85, 195)	119 (79, 178)	125 (82, 182)	130 (92, 197)	142 (93, 221)	< 0.001	< 0.001
Glucose (mg/dL)	116 (99, 159)	106 (96, 131)	114 (99, 154)	122 (100, 163)	127 (101, 178)	< 0.001	< 0.001
Urine creatinine (mg/dL)	92.3 (59.4, 144.0)	142.1 (104.1, 189.4)	98.7 (68.6, 147.7)	80.7 (56.1, 121.3)	62.5 (45.6, 90.3)	< 0.001	< 0.001
uPCR (mg/g)	310 (101, 1,608)	69 (55, 85)	153 (118, 216)	634 (396, 1,110)	3828 (2231, 6827)	< 0.001	< 0.001
uACR (mg/g)	86 (13, 757)	7 (4, 19)	30 (11, 89)	254 (77, 674)	2167 (853, 4219)	< 0.001	< 0.001
uAPR (%)	30.0 (10.8, 57.5)	11.4 (6.3, 25.4)	21.3 (8.6, 42.5)	44.4 (20.0, 64.4)	57.3 (37.4, 66.9)	< 0.001	< 0.001
uNAP (mg/g)	186 (80, 683)	58 (47, 68)	112 (94, 140)	340 (246, 483)	1575 (1019, 2670)	< 0.001	< 0.001

*ACEI* angiotensin-converting-enzyme inhibitors, *ARBs* angiotensin receptor blockers, *CKD* chronic kidney disease, *eGFR* estimated glomerular filtration rate, *NSAIDs* nonsteroidal anti-inflammatory drugs, *OAD* oral antidiabetic agents, *uPCR* urine protein-to-creatinine ratio, *uACR* urine albumin-to-creatinine ratio, *uAPR* urine albumin-to-protein ratio, *uNAP* urine non-albumin proteinuria.

^†^P-values are calculated by Kruskal–Wallis test for continuous variables and Chi-square test for categorical variables.

^‡^P-values for trend are calculated by Spearman's correlation for continuous variables and by Cochran-Armitage trend test for binary variables.

### Risk association among uPCR, uACR, uAPR, uNAP, and all-cause mortality

Over 12,390.79 person-years of follow-up, 657 deaths occurred. Incident all-cause mortality was 53.0 per 1000 person-years. For each doubling increase in uPCR, uACR, and uNAP, the adjusted hazard ratios (aHRs) of all-cause mortality were 1.29 (95% CI 1.24–1.35), 1.12 (1.09–1.16), and 1.41 (1.34–1.49), respectively, in the entire study population. For each 10% increase in uAPR, the aHR was 1.02 (95% CI 0.98–1.06); the effect size (aHR 0.84, 95% CI 0.80–0.88) was similar after additional adjustment for uPCR. The corresponding aHRs of all-cause mortality of uPCR, uACR, uAPR (per doubling increase), and uNAP were 1.30, 1.19, 1.11, and 1.38, respectively, for patients in the concordance group and 1.57, 1.07, 0.83, and 1.61, respectively, for patients in the non-albumin-predominant proteinuria group (Table 4). The findings were similar when changing time scale from age to time-on-study (Supplementary Table S3).

**Table 4:** Hazard ratios (95% confidence interval) of risk of all-cause mortality by uPCR, uACR, uAPR, and uNAP by the concordance between uPCR and uACR and uAPR above or below 40% (based on imputation dataset).

	N	Case	Crude model (95% CI)	Model 1 (95% CI)	Model 2 (95% CI)	Model 3 (95% CI)
**Overall**						
uPCR (per double increase)	2904	657	1.42 (1.37, 1.47)	1.42 (1.37, 1.47)	1.43 (1.38, 1.48)	1.29 (1.24, 1.35)
uACR (per double increase)			1.22 (1.19, 1.25)	1.22 (1.19, 1.25)	1.23 (1.19, 1.26)	1.12 (1.09, 1.16)
uAPR (per 10% increase)			1.09 (1.05, 1.12)	1.08 (1.05, 1.12)	1.08 (1.05, 1.12)	1.02 (0.98, 1.06)
uNAP (per double increase)			1.56 (1.50, 1.62)	1.56 (1.50, 1.63)	1.57 (1.51, 1.63)	1.41 (1.34, 1.49)
**Concordance proteinuria**						
uPCR (per double increase)	2383	497	1.43 (1.37, 1.48)	1.43 (1.37, 1.49)	1.44 (1.38, 1.50)	1.30 (1.24, 1.37)
uACR (per double increase)			1.28 (1.24, 1.32)	1.28 (1.24, 1.32)	1.28 (1.24, 1.32)	1.19 (1.14, 1.23)
uAPR (per 10% increase)			1.19 (1.15, 1.24)	1.19 (1.14, 1.24)	1.19 (1.15, 1.24)	1.11 (1.06, 1.16)
uNAP (per double increase)			1.54 (1.47, 1.61)	1.54 (1.47, 1.61)	1.55 (1.48, 1.62)	1.38 (1.30, 1.46)
**Non-albumin predominant proteinuria**						
uPCR (per double increase)	363	150	1.69 (1.48, 1.92)	1.68 (1.46, 1.93)	1.70 (1.47, 1.96)	1.57 (1.32, 1.87)
uACR (per double increase)			1.15 (1.05, 1.26)	1.15 (1.05, 1.26)	1.16 (1.05, 1.27)	1.07 (0.96, 1.19)
uAPR (per 10% increase)			0.83 (0.71, 0.97)	0.84 (0.72, 0.99)	0.85 (0.72, 1.00)	0.83 (0.70, 0.98)
uNAP (per double increase)			1.70 (1.50, 1.93)	1.70 (1.49, 1.95)	1.73 (1.50, 1.98)	1.61 (1.36, 1.90)
**uAPR < 40%**						
uPCR (per double increase)	1683	320	1.77 (1.67, 1.87)	1.76 (1.66, 1.88)	1.77 (1.66, 1.88)	1.64 (1.51, 1.79)
uACR (per double increase)			1.44 (1.36, 1.51)	1.44 (1.36, 1.51)	1.44 (1.36, 1.52)	1.25 (1.17, 1.34)
uNAP (per double increase)			1.79 (1.69, 1.90)	1.79 (1.69, 1.91)	1.79 (1.69, 1.91)	1.67 (1.54, 1.82)
**uAPR ≥ 40%**						
uPCR (per double increase)	1221	337	1.42 (1.33, 1.51)	1.42 (1.33, 1.51)	1.43 (1.34, 1.52)	1.27 (1.18, 1.37)
uACR (per double increase)			1.37 (1.29, 1.45)	1.37 (1.29, 1.45)	1.38 (1.30, 1.47)	1.24 (1.16, 1.33)
uNAP (per double increase)			1.45 (1.37, 1.55)	1.45 (1.36, 1.55)	1.46 (1.37, 1.56)	1.28 (1.19, 1.39)

Model 1: Adjusted gender, diabetes, hypertension, cardiovascular disease and cancer.

Model 2: Adjusted gender, diabetes, hypertension, cardiovascular disease, cancer, ACEI, ARBs, and anti-platelet agents.

Model 3: Adjusted gender, diabetes, hypertension, cardiovascular disease, cancer, ACEI, ARBs, anti-platelet agents, eGFR, hemoglobin, and glucose.

*ACEI* angiotensin-converting-enzyme inhibitors, *ARBs* angiotensin receptor blockers, *eGFR* estimated glomerular filtration rate, *uAPR* urine albumin-to-protein ratio.

**Figure 2:**
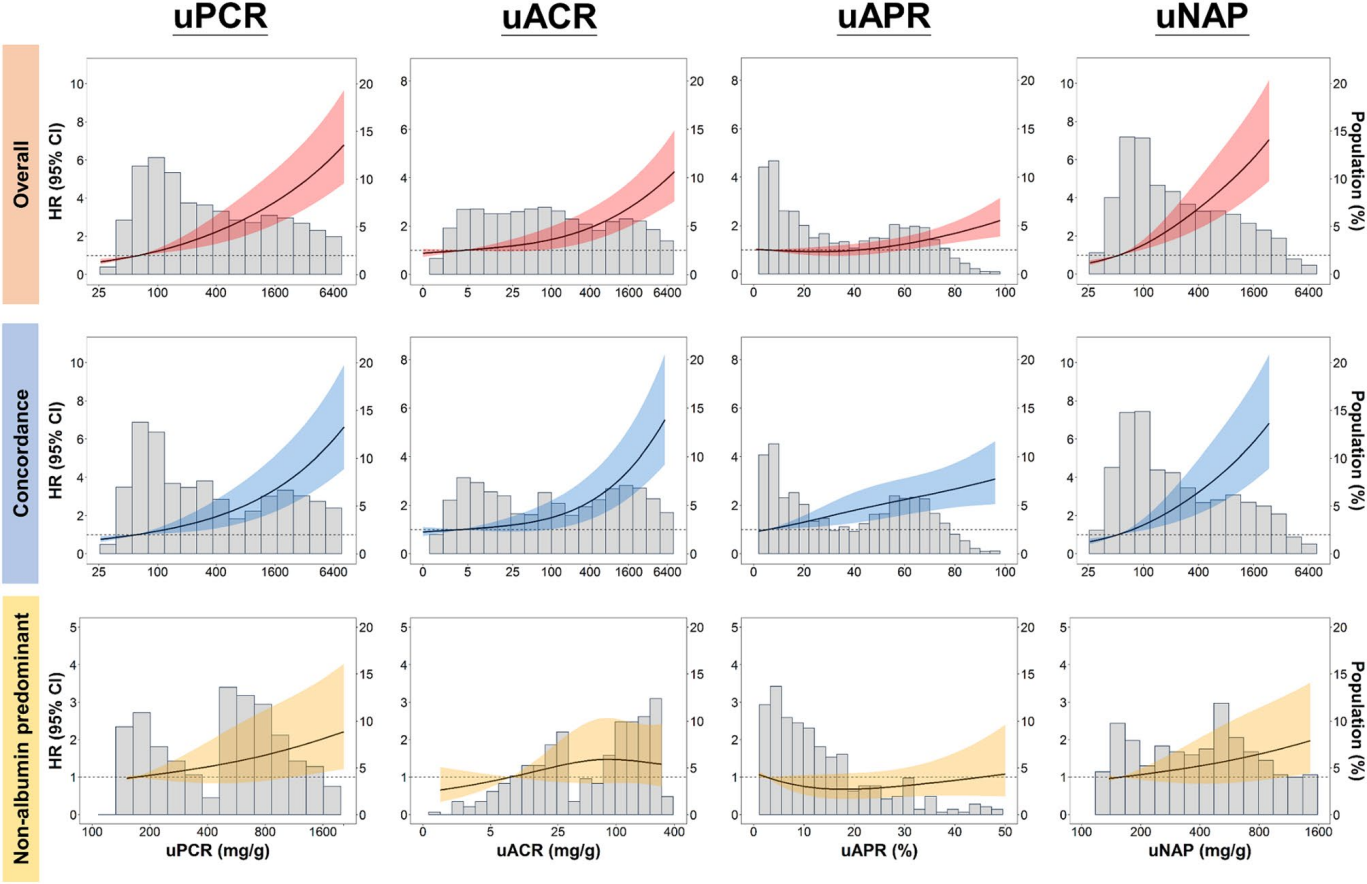
Hazard ratios for all-cause mortality based on uPCR, uACR, uAPR, and uNAP. Solid black lines represent aHRs based on restricted cubic splines for each urinary biomarker with knots at the 10th, 50th, and 90th percentiles. Dark-red shaded areas represent 95% CI. The reference was set at the 10th percentile of each urinary biomarker.

A continuous dose–response association was observed between uPCR, uACR, and uNAP and all-cause mortality in patients in the concordant and non-albumin-predominant proteinuria groups (Fig. 2). Only uPCR and uNAP demonstrated a linear-appearing dose–response relationship with all-cause mortality (Fig. 2). For the continual association between uACR and all-cause mortality for the non-albumin-predominant proteinuria group, we did not find any significant association of uACR with all-cause mortality (Fig. 2). In the concordant group, the continuous risk shape between uACR and all-cause mortality was similar to that of uPCR with a lower risk plateau point at about 100 mg/mg creatinine (Fig. 2). Among patients in the concordant group, elevated uAPR increased the risk of all-cause mortality (Fig. 2) with a linear-appearing dose–response curve. When plotting dose–response curves of uPCR, uACR, uAPR, and uNAP with all-cause mortality in each concordant subgroup with different severity, uPCR, uACR and uNAP in patients with concomitant severe uPCR and uACR showed a continuous significant risk of death when using per doubling increase level of uPCR or uNAP of this subgroup as the reference (Supplementary Fig. S2).

**Figure 3:**
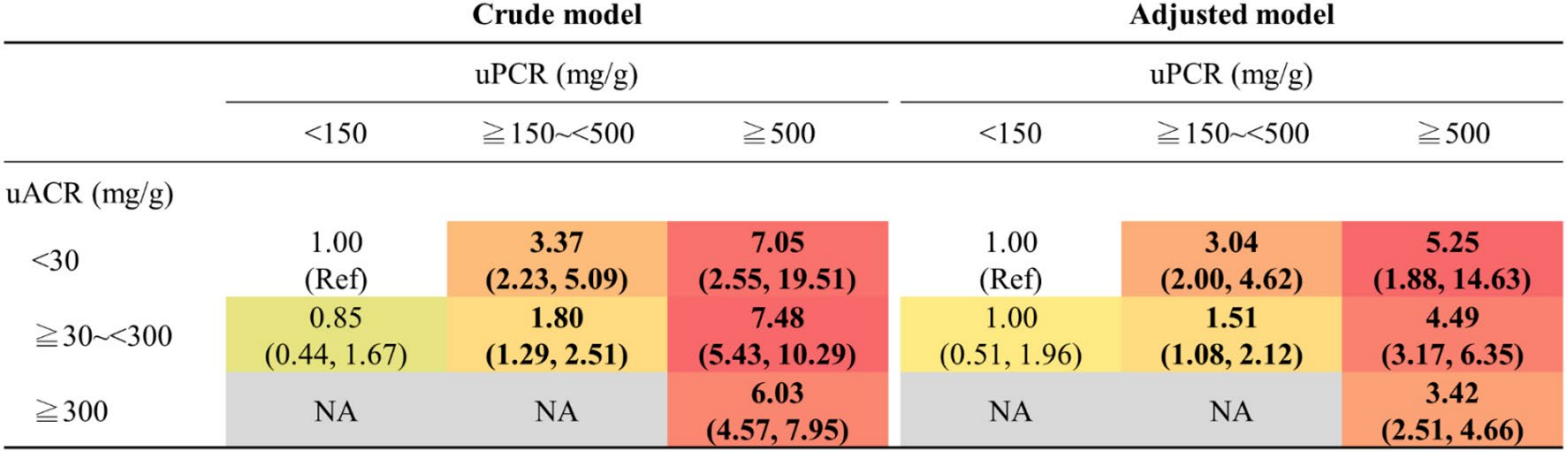
Risk matrices demonstrating aHRs for all-cause mortality according to the concordant pattern among categories of uPCR (< 150, ≥ 150 to < 500, and ≥ 500 mg/g creatinine) and uACR (< 30, ≥ 30 to < 300, and ≥ 300 mg/g creatinine). The color of the reference cells and cells with a risk estimate equal to 1.0 is white, which stands for the group with uPCR < 150 mg/g creatinine and uACR < 30 mg/g creatinine. For a hazard ratio below 1.0, we used green color, whereas for a hazard ratio above 1.0, we used red. We colored the cells from light (close to 1.0) to dark (toward risk or protective) based on the range of the risk estimates of each 3 × 3 table. Cells without risk estimates (NA) are colored gray. The numbers in bold indicate that they are significant (*P* < 0.05).

The 3 × 3 risk matrices using normal, moderate, and severe categories of uPCR combined with uACR categories (normal, microalbuminuria, and macroalbuminuria) revealed that patients in the severe category of uPCR and the concomitant macroalbuminuria category exhibited the highest risk of all-cause mortality (aHR 3.42, 95% CI 2.51, 4.66) among patients in the concordant group (main diagonal groups) (Fig. 3). Among patients with non-albumin-predominant proteinuria, those with severe proteinuria and uACR < 30 mg/g had the highest risk of all-cause mortality (aHR 5.25, 95% CI: 1.88, 14.63) (Fig. 3). We did not find any interaction between the subgroups of the concordant group and age, sex, diabetes, hypertension, CVD, CKD, and uAPR (cut-off = 40%) (Supplementary Fig. S3). By contrast, we found that the cut-offs of uAPR setting at 30% or 40% significantly modified the risk association of uPCR or uNAP with all-cause mortality. Notably, the risk association of uPCR and uNAP on mortality significantly differentially increased among those with uAPR below 30% or 40% compared with those above these cut-offs (Supplementary Table S4).

Regarding risk prediction, uNAP [Harrell’s c statistic: 0.834 (95% CI 0.819, 0.849)] significantly improved the discriminative performance compared to that of uPCR [0.828 (0.812, 0.843), *P* = 0.03] or uACR [0.817 (0.800, 0.833), *P* = 0.004] (Supplementary Fig. S4). The optimal cut-off values for the prediction of all-cause mortality were 269.4, 60.3, and 262.6 mg/g creatinine for uPCR, uACR, and uNAP, respectively, in the overall population. Among concordance groups, the corresponding cut-off values were 818.9, 211.5, and 337.9 mg/g creatinine (Supplementary Fig. S5a). When stratifying by age, the optimal cutoff for uPCR became much higher to 479.7 mg/g creatinine among patients 65 years of age or older whereas it remains the same at 264.5 mg/g creatinine for uNAP (Supplementary Fig. S5b). By sex, the optimal cut-off value of uPCR for males was much higher than that for females (455.8 vs. 309.2 mg/g creatinine). By contrast, the optimal cut-off value of uNAP for males was much lower than that for females (262.6 vs. 384.4 mg/g creatinine) (Supplementary Fig. S5c).

## Discussion

Concurrent measurements of uACR and uPCR to derive uNAP improve the risk assessment of all-cause mortality among patients with clinically indicated urinary protein quantification, including diabetes, hypertension, and CKD. In the uPCR and uACR concordant group, the association of uPCR, uACR, or uNAP with deaths followed a dose–response relationship. However, in the subgroup of the concordant group, uPCR, uACR, and uNAP were associated with all-cause mortality only in patients with concomitant severe proteinuria and macroalbuminuria. Notably, uNAP demonstrated the most linear-appearing dose–response relationship with all-cause mortality and significantly improve the prognostic performance for mortality compared with those using uPCR or uACR. In the non-albumin-predominant group, patients with severe proteinuria and normal albuminuria had the highest mortality risk compared with those without proteinuria and albuminuria. More importantly, the risk association of uPCR or uNAP with all-cause mortality was considerably more significant among patients with uAPR < 40%, which indicates to the patient having tubulointerstitial injury-dominant kidney disease or other non-renal systemic diseases associated with overproduction of low-molecular-weight proteins, such as immunoglobulin light chains in plasma cell dyscrasias.

We confirmed the prospective association between uPCR or uNAP and all-cause mortality, particularly significant among patients with uAPR < 40%. The underlying mechanisms that make tubular protein-dominant proteinuria a significant risk marker of mortality remains unknown. Conventionally, tubular proteinuria is defined as the free filtration of low-molecular-weight proteins across the glomerulus, which are typically reabsorbed in the proximal tubule and appear in the urine owing to proximal tubular injury or protein overload because of excess production. Traditional urinary tubular biomarkers include α1-microglobulin, β2-microglobulin (b2-MG), retinol-binding protein (RBP), and *N*-acetyl-β-glucosaminidase (NAG), whereas emerging urinary tubular markers such as neutrophil gelatinase-associated lipocalin (NGAL) and kidney injury molecule-1 (KIM-1) have been used to detect acute kidney injury (AKI)^19^. Few studies have focused on the prognostic value of these urinary tubular markers in predicting mortality in the general population. In several Cadmium-polluted areas in Japan, b2-MG or RBP has been linked to mortality since the 1990s^20^. Recently, Suwazono et al. determined that kidney tubular dysfunction quantified by the level of b2-MG and NAG was significantly associated with mortality in a non-cadmium polluted area^21^. On the other hand, few studies have evaluated the association between urinary biomarkers of AKI and CKD progression on mortality. For instance, Lobato et al. observed that urinary NGAL was independently associated with rapid progression to ESRD and death in patients with advanced CKD^22^. However, Seibert et al. did not observe any significant association of urinary NGAL and KIM-1 with CKD progression^23^. In the Health Aging and Body Composition (Health ABC) study, Sarnak et al. first observed the independent effect of KIM-1 on the risk of all-cause mortality^24^. Moreover, in patients with diabetic nephropathy, KIM-1 was found to be independently associated with CKD progression and all-cause mortality^25^. Despite the existing evidence that supports the potential utility of urinary tubular biomarkers in the risk assessment of mortality, the cost-effectiveness issues related to measuring a specific tubular protein poses a hindrance in daily clinical practice. Another potential mechanism underlying the elevated uNAP is related to non-renal systemic disease, particularly cancer and autoimmune disorders, and can offer a potential possibility to expand the clinical utility of uNAP. For instance, IgG-uria has been determined to be associated with tubular injury, indicated by increased excretion of α1-microglobulin in patients with glomerular diseases^26^. Also, in vitro studies have revealed that high-molecular-weight plasma proteins are associated with increased apoptosis of proximal tubular cells via the Fas/Fas ligand pathway or impaired tubule megalin expression^27,28^. Therefore, future research should clarify whether uNAP can serve as a favorable risk marker of protein loading and tubular injuries and systematically evaluate the prognostic role of uNAP in various clinical outcomes, such as AKI, CKD, immune disorders, and malignancies.

Our approach of directly measuring uNAP by subtracting uACR from uPCR provides a practical solution to mortality risk stratification. First, compared with the effect sizes of uPCR and uACR on the risk of deaths in the overall population, the effect size of uNAP was significantly greater. The predictive performance of uNAP for mortality is also significantly better than uPCR or uACR from the discrimination perspectives and with comparable calibration performance. The most apparent linear relationship between uNAP and all-cause mortality substantiates that uNAP can serve as an independent marker of mortality and may be more sensitive than traditional uPCR or uACR. Second, concomitant measurements of uPCR and uACR are relatively inexpensive and provide quantifiable information regarding the severity of tubular proteinuria. However, very few studies have evaluated the clinical significance of uNAP in large population studies. The first population prevalence study conducted in 2016 in the US showed that the prevalence of significant uNAP was approximately 10–20%^10^. The authors also pointed out that screening only for albuminuria may miss 40% of patients with significant uNAP. However, this study used a semi-quantitative approach to define significant uNAP, rather than a numerical measurement^10^. Recently, Kwon et al. used the same definition as our study to quantify uNAP and determined that this parameter was significantly associated with tubulointerstitial inflammation among patients with lupus nephritis^13^. More importantly, high uNAP was associated with inadequate response to immunosuppressive agents^13^. Therefore, further mechanistic and clinical research is required to verify the pathogenesis of tubular proteinuria, the cut-off value of uNAP, and the corresponding therapeutic strategies in heterogeneous populations. In the present study, we found uNAP have a comparable prognostic cut-off value at 260–270 mg/g creatinine with uPCR for all-cause mortality and this threshold is less influenced by proteinuric classifications, age, and sex. This characteristic is of particular importance in clinical practice. Third, routine screening of uNAP can enable clinicians to discern patients’ environmental risk exposures, such as drugs, toxicants, metals, and even air pollution. This approach would be particularly useful for patients with CKD because heavy metals, such as cadmium, lead, arsenic, and mercury, are associated with tubulointerstitial injuries primarily arising from apoptosis, inflammation owing to oxidative stress in the proximal convoluted tubules, and mitochondrial dysfunction^29^. Drug-induced acute tubulointerstitial nephritis is also a crucial etiology of tubular proteinuria; for example, NSAID-related hypersensitivity and the formation of immune complexes may attack renal tubules^30^. However, the correlations between uNAP and various environmental factors remain unexplored.

Nonetheless, our study had several limitations. First, we could not affirm the causal relationship between uNAP and all-cause mortality owing to the observational nature of the present study. Notwithstanding, the current study findings pave the way for future research to explore the clinical significance of uNAP in a more comprehensive manner. Second, residual confounding could not be entirely excluded because we did not have detailed information regarding patients’ lifestyle and environmental exposures. Therefore, clarifying whether cumulative environmental hazards can be approximated using uNAP and determining its association with population attributable risk of mortality are top research priorities. Third, misclassification bias could not be completely excluded because we relied on a single measurement of uNAP. Among 724 patients with available uNAP, we observed that the intraclass correlation coefficient was 0.664 showing relative stability of uNAP over time.

In conclusion, our study findings advocate for the simultaneous measurement of uPCR and uACR in daily clinical practice. Both uAPR and uNAP can be easily derived at a reasonable cost and facilitate a more accurate mortality risk assessment by classifying the origin of proteinuria. Nevertheless, further research is required to verify the uniqueness of uAPR and uNAP in predicting various adverse clinical outcomes in different populations.

## Methods

### Study population

In 2017, the Big Data Center and the Office of Information Technology of China Medical University Hospital (CMUH) established the CMUH Clinical Research Data Repository (CRDR), which carefully verified and validated data from various clinical sources to unify patient information generated during the healthcare process such that it can be tracked. Between January 1, 2003, and December 31, 2017, the CMUH-CRDR documented the details of 2,712,350 patients who had sought care at CMUH. Patient information included data on administration and demography, diagnosis, medical and surgical procedures, prescriptions, laboratory measurements, physiological monitoring data, hospitalization, and status of catastrophic illness. The interoperability of the CMUH-CRDR has further expanded access to national population-based health-related databases (e.g., the mortality database), which are systematically maintained by the Health and Welfare Data Science Center (HWDC) of the Ministry of Health and Welfare (MOHW). All patients enrolled in the CMUH-CRDR were followed up until December 31, 2017, or death—whichever occurred earlier. The description of CMUH-CRDR had been reported in our previous work^31,32^.

The present study cohort comprised patients aged between 18 and 90 years who had concurrent uACR and uPCR measurements from the same urine specimen in both inpatient and outpatient settings from January 2003 to June 2017. The index date was defined as the day on which uACR and uPCR were measured. Supplementary Fig. S6 shows the detailed case selection process. The International Classification of Disease code of comorbidities is given in Supplementary Table S5.

All methods in this study were performed in accordance with the relevant guidelines/regulations. The study was approved by the Big Data Center of China Medical University Hospital and the Research Ethical Committee/Institutional Review Board of China Medical University Hospital (CMUH105-REC3-068) and the need to obtain informed consent for the present study was waived by the Research Ethical Committee of China Medical University Hospital.

### Measurement of serum creatinine, uACR, uPCR, uAPR, and uNAP

Serum creatinine levels were measured using the Jaffe rate method (kinetic alkaline picrate) at CMUH Central Laboratory by using a Beckman UniCel DxC 800 immunoassay system (Beckman Coulter Inc., Brea, CA, USA). The estimated glomerular filtration rate (eGFR) was calculated using the abbreviated Chronic Kidney Disease Epidemiology Collaboration (CKD-EPI) equation (eGFR = 141 × min(Scr/κ, 1)^α^ × max(Scr/κ, 1)^−1.209^ × 0.993^Age^ × 1.018 [if female] × 1.159 [if black])^33^. Serum creatinine levels at enrollment were used to define the baseline eGFR and corresponding CKD stages using the following cut-off values: > 90, 60–89.9, 30–59.9, 15–29.9, and < 15 mL/min/1.73 m^2^. uACR and uPCR were calculated as the ratio of urine albumin and protein to creatinine (mg/g creatinine), respectively. Urinary albumin concentration was determined using a turbidimetric method (Microalbumin reagent, Synchron System, Beckman Coulter). Urinary protein concentration was quantified through a timed endpoint method (Microprotein reagent, Synchron System, Beckman Coulter). The urinary creatinine level was measured using the Jaffe rate method (CREm reagent, Synchron System, Beckman Coulter). uAPR was derived by dividing uACR by uPCR, whereas uNAP was calculated by subtracting uACR from uPCR.

### Clinical data captured through CMUH data warehouse and national dataset tracking

Sociodemographic variables and baseline comorbidities and medications were determined based on the information obtained from the CMUH-CRDR within a 1-year window before the index date, whereas an additional 3-month inclusion window after the index date was applied to biochemical measures. If patients did not have any visit records and medication records within 1 year prior to the index date, they were treated as having missing information on comorbidities and medications, respectively. The number of patients with available information for the study variables is provided in Supplementary Table S6. The diagnosis of cancer was determined if the patient had a catastrophic illness certificate for malignancy. The catastrophic illnesses are defined by Taiwan’s National Health Insurance program, and patients who are diagnosed with a catastrophic illness are exempted from copayments. Dates of death were verified at the National Death Registry of the MOHW of Taiwan. To replace the missing values, we performed multiple imputations using an iterative Markov chain Monte Carlo procedure with 20 imputations and 100 iterations. We used the data from the multiple imputations in the subsequent multivariable analyses.

### Statistical analysis

Continuous variables were expressed as medians and interquartile ranges (IQRs) and compared using the nonparametric Kruskal–Wallis test, whereas categorical variables were expressed as frequency (percentage) and compared using the chi-square test. Due to strong right skewness, values of uPCR, uACR, and uNAP were log-transformed of base 2 for analysis as continuous data. We designed a 3 × 3 classification matrix based on severity grades of both uACR (normal: < 30; microalbuminuria: 30 to < 300; macroalbuminuria: ≥ 300 mg/g creatinine) and uPCR (normal: < 150; moderate: 150 to < 500; severe: ≥ 500 mg/g creatinine) and categorized patients into 3 groups as follows: (1) concordant proteinuria, (2) non-albumin-predominant proteinuria (upper diagonal of the matrix), and (3) albumin-predominant proteinuria (lower diagonal of the matrix) (Supplementary Fig. S1). We also compared patients’ characteristics by using the uAPR and uNAP quartiles. The associations between uPCR, uACR, uAPR, and uNAP modeled as both continuous and categorical exposures and risk of all-cause mortality were estimated using multivariable Cox regression analysis. The time scale for survival analysis was age, and the late entry method was applied using age at baseline as the individual entry time to align risk sets appropriately. The exit time was the date of death or the administrative censoring date of December 31, 2017. Multivariable Cox regression models were initially adjusted for sociodemographic and lifestyle variables, such as sex, diabetes, hypertension, cardiovascular diseases (CVD, including coronary artery disease, heart failure, and stroke), and cancer diagnosis; then adjusted for medications such as angiotensin-converting enzyme inhibitors (ACEIs), angiotensin II receptor blockers (ARBs), and antiplatelet agents; and finally adjusted for biochemical measures, including eGFR, serum albumin, and hemoglobin. We further characterized the dose–response association with all-cause mortality by using a restricted cubic spline model with 3 knots located at the 10th, 50th, and 90th percentiles of the overall distribution for uACR, uPCR, uAPR, and uNAP in each main group. We further mapped the risk of all-cause mortality in the classification matrix using patients with normal uACR and uPCR as the reference group. We performed exploratory subgroup analysis to evaluate effect modification among patients with a concordant albuminuria-proteinuria status based on age, sex, diabetes, hypertension, CKD, and uAPR with a cut-off of 40%. To compare the prognostic performance of uACR, uPCR, uAPR, and uNAP for all-cause mortality, we applied Harrell’s C-Statistic to deal with the time-dependent receiver operating characteristics (ROC) curve for right-censored survival data^34,35^. The fully adjusted model served as the reference for the prognostic performance of new models incorporating either of these proteinuria markers. We also plotted the observed vs predicted risk probability to show the differences in calibrations of all risk models for all-cause mortality. To define the prognostic threshold in predicting all-cause mortality, optimal cut-off values were determined for all proteinuria markers in overall, concordant, and non-albumin proteinuria groups when the log-rank test statistics was maximal^36^. All statistical analyses were performed using SAS version 9.4 (SAS Institute Inc., Cary, NC, USA) and R version 3.5.1 (R Foundation for Statistical Computing, Vienna, Austria). The 2-sided statistical significance level of α was set at 0.05.

### Ethical approval

The study was approved by the Research Ethical Committee/Institutional Review Board of China Medical University Hospital (CMUH105-REC3-068).

## Supplementary Information

Supplementary Information.

## Data Availability

The datasets analyzed during the current study are not publicly available due to them containing information that could compromise research participant privacy but are available from the corresponding author, CCK, on reasonable request.
