# Supplementary material for: The ratio and difference of urine protein-to-creatinine ratio and albumin-to-creatinine ratio facilitate risk prediction of all-cause mortality
Source: Sci Rep. 2021 Apr 12;11:7851. doi: 10.1038/s41598-021-86541-3 (PMC8041921; doi:10.1038/s41598-021-86541-3)

**The Ratio and Difference of Urine Protein-to-Creatinine Ratio and Albumin-to-Creatinine Ratio Facilitate Risk Prediction of All-cause Mortality**

David Ray Chang, MD^1^, Hung-Chieh Yeh, MD^1^, I-Wen Ting, MD^1^, Chen-Yuan Lin, MD, PhD^2^, Han-Chun Huang, MS^3^, Hsiu-Yin Chiang, PhD^3^, Shih-Ni Chang, MS^3^, Hsiu-Chen Tsai, MS^3^, Yen-Chun Lo, MS^3^, Chiung-Tzu Hsiao, MT^4,5^, Pei-Lun Chu, MD, PhD^6^,
Chin-Chi Kuo, MD, PhD*^1,3^

*Corresponding author

^1^ Division of Nephrology, Department of Internal Medicine, China Medical University Hospital and College of Medicine, China Medical University, Taichung, Taiwan

^2^ Division of Hematology and Oncology, Department of Internal Medicine, China Medical University Hospital and College of Medicine, China Medical University, Taichung, Taiwan

^3^ Big Data Center, China Medical University Hospital and College of Medicine, China Medical University, Taichung, Taiwan

^4^ Department of Laboratory Medicine, China Medical University Hospital, Taichung, Taiwan

^5^ Department of Medical Laboratory Science and Biotechnology, China Medical University, Taichung, Taiwan

^6^ Division of Nephrology, Department of Internal Medicine, Fu-Jen Catholic University Hospital and College of Medicine, School of Medicine, Fu-Jen Catholic University, New Taipei City, Taiwan

**Corresponding author:** Chin-Chi Kuo, MD, PhD.

Big Data Center, China Medical University Hospital and College of Medicine, China Medical University, Taichung, Taiwan

**Address:** 2, Yude Rd., North Dist., Taichung City 404

**Tel:** 886-4-22052121 ext. 2910

**Email:** chinchik@gmail.com

**Supplementary Material**

**Table S1**. Baseline demographic and clinical characteristics stratified by quartiles of urine albumin-to-creatinine ratio (uACR).

**Table S2**. Baseline demographic and clinical characteristics stratified by quartiles of urine protein-to-creatinine ratio (uPCR).

**Table S3.** Hazard Ratios (95% confidence interval) of risk of all-cause mortality by uPCR, uACR, uAPR, and uNAP by the concordance between uPCR and uACR and uAPR above or below 40% (based on imputation dataset and model with time-on-study as the time-scale).

**Table S4**. Hazard ratios (HRs) and 95% CIs of uPCR, uACR, and uNAP for all-cause mortality by uAPR at different cut-off values from 30% to 70%. Adjustment factors were the same as those for Model 3 in Table 4.

**Table S5**. The international classification of disease of comorbidities.

**Table S6.** The number of patients with available information for the baseline demographic and clinical characteristics in the study population (n = 2904 patients).

**Figure S1.** The classification matrix base on severity grades of both uACR and uPCR and categorized patients into three group (concordance proteinuria, non-albumin predominant proteinuria, and albumin predominant proteinuria).

**Figure S2.** Hazard ratios (HRs) for all-cause mortality according to uPCR, uACR, uAPR, and uNAP in each concordant proteinuria. Solid lines represent adjusted HRs based on restricted cubic splines for each urinary biomarker with knots at the 10th, 50th, and 90th percentiles. Shaded areas represent 95% confidence interval. The reference was set at the 10th percentile of each urinary biomarker. The blue, green and dark-red lines and shaded areas represent the concordant subgroups from uPCR <150 mg/g creatinine and uACR <30 mg/g creatinine to uPCR ≧500 mg/g creatinine and uACR ≧300 mg/g creatinine, respectively.

**Figure S3.** Hazard ratios (HRs) and 95% CIs for the risk of all-cause mortality comparing the moderate (150≦uPCR <500 mg/g creatinine and 30≦uACR <300 mg/g creatinine) and server (creatinine to uPCR ≧500 mg/g creatinine and uACR ≧300 mg/g creatinine) verse normal (uPCR <150 mg/g creatinine and uACR <30 mg/g creatinine) in concordant proteinuria, by patient characteristics at baseline. Adjustment factors were the same as those for Model 3 in Table 4.

**Figure S4.** Discrimination statistics (based on an imputed database) and calibration plots (based on an actual database with the outcome of 3-year all-cause mortality) for reference (Model 3 in Table 4) and new models (Model 3 + uNAP, Model 3 + uPCR, Model 3 + uACR, and Model 3 + uAPR).

**Figure S5a.** Cut-off determination of uPCR, uACR, uAPR, and uNAP for all-cause mortality by patients’ proteinuric classifications**.** uPCR, urine protein-to-creatinine ratio; uACR, urine albumin-to-creatinine ratio; uAPR, urine albumin-to-protein ratio; uNAP, urine non-albumin proteinuria.

**Figure S5b.** Cut-off determination of uPCR, uACR, uAPR, and uNAP for all-cause mortality by age cutoff of 65 years**.** uPCR, urine protein-to-creatinine ratio; uACR, urine albumin-to-creatinine ratio; uAPR, urine albumin-to-protein ratio; uNAP, urine non-albumin proteinuria.

**Figure S5c.** Cut-off determination of uPCR, uACR, uAPR, and uNAP for all-cause mortality by patients’ sex**.** uPCR, urine protein-to-creatinine ratio; uACR, urine albumin-to-creatinine ratio; uAPR, urine albumin-to-protein ratio; uNAP, urine non-albumin proteinuria.

**Figure S6.** Flow diagram of patient selection.

**Table S1**. Baseline demographic and clinical characteristics stratified by quartiles of urine albumin-to-creatinine ratio (uACR).

| **Variable** | **Total (n= 2904)** | **uACR≦13 (n= 733)** | **13 < uACR≦86 (n= 718)** | **86 < uACR≦757 (n= 727)** | **uACR>57.5 (n= 726)** | **P-value**† | **P for trend**‡ |
| --- | --- | --- | --- | --- | --- | --- | --- |
| Age (year), median (IQR) | 58.6 (45.4, 70.8) | 50.9 (37.2, 62.3) | 59.8 (47.4, 72.7) | 63.4 (52.0, 74.8) | 59.9 (47.1, 71.2) | <0.001 | <0.001 |
| Female, n (%) | 1697 (58.4) | 453 (61.8) | 416 (57.9) | 428 (58.9) | 400 (55.1) | 0.08 | 0.02 |
| Body mass index (kg/m^2^), median (IQR) | 24.1 (21.8, 27.3) | 23.9 (21.3, 26.5) | 24.0 (21.5, 26.8) | 23.9 (22.1, 27.4) | 24.5 (22.0, 27.9) | 0.03 | 0.003 |
| Follow-up duration (year), median (IQR) | 3.32 (1.41, 6.28) | 3.52 (1.85, 6.31) | 3.69 (1.47, 6.86) | 2.9 (1.1, 6.01) | 2.92 (1.15, 5.75) | <0.001 | <0.001 |
| **Baseline comorbidities, n (%)** |  |  |  |  |  |  |  |
| CKD stage, 1-2 | 1343 (47.3) | 563 (80.5) | 373 (53.2) | 251 (35.1) | 156 (21.6) | <0.001 | <0.001 |
| CKD stage, 3-5 | 1495 (52.7) | 136 (19.5) | 328 (46.8) | 464 (64.9) | 567 (78.4) |  |  |
| Diabetes | 928 (32.1) | 123 (16.9) | 214 (29.9) | 253 (34.9) | 338 (46.6) | <0.001 | <0.001 |
| Hypertension | 1245 (43.0) | 158 (21.7) | 289 (40.4) | 349 (48.2) | 449 (61.8) | <0.001 | <0.001 |
| Cardiovascular disease | 766 (26.5) | 107 (14.7) | 185 (25.9) | 223 (30.8) | 251 (34.7) | <0.001 | <0.001 |
| Cancer before enrollment | 265 (9.13) | 45 (6.10) | 81 (11.3) | 75 (10.3) | 64 (8.80) | 0.004 | 0.13 |
| Cancer after enrollment | 166 (5.72) | 30 (4.10) | 50 (7.00) | 52 (7.20) | 34 (4.70) | 0.02 | 0.60 |
| Cancer (before or after enrollment) | 431 (14.8) | 75 (10.2) | 131 (18.2) | 127 (17.5) | 98 (13.5) | <0.001 | 0.12 |
| **Baseline medication profiles, n (%)** |  |  |  |  |  |  |  |
| Pentoxifylline | 389 (14.8) | 31 (5.20) | 77 (11.7) | 130 (19.3) | 151 (21.6) | <0.001 | <0.001 |
| Dipyridamole | 91 (3.47) | 9 (1.50) | 18 (2.70) | 30 (4.50) | 34 (4.90) | 0.003 | <0.001 |
| Anti-platelet agents | 683 (26.1) | 116 (19.4) | 159 (24.2) | 200 (29.8) | 208 (29.8) | <0.001 | <0.001 |
| NSAIDs | 721 (27.5) | 175 (29.2) | 190 (28.9) | 190 (28.3) | 166 (23.8) | 0.09 | 0.03 |
| Contrast | 479 (18.2) | 106 (17.7) | 137 (20.9) | 130 (19.3) | 106 (15.2) | 0.04 | 0.15 |
| Antihypertension agents |  |  |  |  |  |  |  |
| ACEI | 465 (17.7) | 44 (7.30) | 103 (15.7) | 121 (18.0) | 197 (28.2) | <0.001 | <0.001 |
| ARBs | 919 (35.0) | 106 (17.7) | 224 (34.1) | 252 (37.5) | 337 (48.3) | <0.001 | <0.001 |
| Diuretics | 1192 (45.4) | 166 (27.7) | 250 (38.1) | 310 (46.1) | 466 (66.8) | <0.001 | <0.001 |
| Antidiabetic agents |  |  |  |  |  |  |  |
| OAD | 724 (27.6) | 101 (16.9) | 183 (27.9) | 191 (28.4) | 249 (35.7) | <0.001 | <0.001 |
| Insulin | 539 (20.5) | 52 (8.70) | 103 (15.7) | 157 (23.4) | 227 (32.5) | <0.001 | <0.001 |
| **Baseline biochemical profiles, median (IQR)** |  |  |  |  |  |  |  |
| eGFR (mL/min/1.73m2) | 56.8 (27.8, 91.8) | 91.7 (67.0, 107) | 63.3 (40.6, 94.1) | 45.3 (22.1, 73.2) | 25.4 (10.1, 55.7) | <0.001 | <0.001 |
| Serum creatinine (mg/dL) | 1.23 (0.86, 2.16) | 0.91 (0.73, 1.15) | 1.12 (0.8, 1.59) | 1.48 (1.01, 2.58) | 2.37 (1.24, 4.91) | <0.001 | <0.001 |
| Blood urea nitrogen (mg/dL) | 20 (13, 36) | 13 (10, 17) | 17 (12, 25) | 24 (15, 41) | 36 (20, 62) | <0.001 | <0.001 |
| Serum uric acid (mg/dL) | 6.5 (5.2, 8.0) | 5.7 (4.8, 7) | 6.1 (5, 7.6) | 6.7 (5.4, 8.3) | 7.4 (6.2, 8.8) | <0.001 | <0.001 |
| Sodium (mmol/L) | 138 (136, 140) | 138 (136, 140) | 138 (136, 140) | 138 (135, 140) | 138 (135, 139) | <0.001 | <0.001 |
| Potassium (mmol/L) | 4.1 (3.7, 4.5) | 4 (3.8, 4.3) | 4 (3.7, 4.4) | 4.1 (3.7, 4.5) | 4.3 (3.8, 4.7) | <0.001 | <0.001 |
| Calcium (mg/dL) | 8.7 (8.2, 9.2) | 9 (8.6, 9.3) | 8.9 (8.4, 9.3) | 8.8 (8.3, 9.3) | 8.4 (7.9, 8.9) | <0.001 | <0.001 |
| Phosphate (mg/dL) | 4.0 (3.4, 4.8) | 3.7 (3, 4.2) | 3.7 (3.2, 4.2) | 3.9 (3.3, 4.6) | 4.4 (3.7, 5.6) | <0.001 | <0.001 |
| Serum albumin (g/dL) | 4.0 (3.3, 4.4) | 4.3 (4, 4.6) | 4.1 (3.6, 4.4) | 4 (3.5, 4.3) | 3.4 (2.8, 3.9) | <0.001 | <0.001 |
| Hemoglobin (g/dL) | 11.9 (9.80, 13.8) | 13.3 (11.9, 14.6) | 12.5 (10.5, 14.05) | 11.4 (9.6, 13.4) | 10.5 (9, 12.8) | <0.001 | <0.001 |
| Total cholesterol (mg/dL) | 181 (152, 214) | 179 (155, 211) | 174 (147, 204) | 180 (150, 208) | 193 (159, 232) | <0.001 | <0.001 |
| Triglyceride (mg/dL) | 129 (85, 195) | 115 (73, 173) | 121 (83, 176) | 130 (89, 195) | 148 (101, 227) | <0.001 | <0.001 |
| Glucose (mg/dL) | 116 (99, 159) | 107 (96, 136) | 119 (99, 159) | 116 (99, 161) | 125.5 (100, 174) | <0.001 | <0.001 |
| Urine creatinine (mg/dL) | 92.3 (59.4, 144) | 136 (94.5, 184) | 98.1 (66.5, 145) | 78.9 (52.5, 119) | 69.0 (48.2, 101) | <0.001 | <0.001 |
| uPCR (mg/g) | 310 (101, 1608) | 71.5 (55.3, 95.9) | 147 (109, 246) | 606 (355, 999) | 3781 (2217, 6583) | <0.001 | <0.001 |
| uACR (mg/g) | 86.1 (12.8, 757) | 5.09 (3.24, 7.86) | 33.9 (21.5, 54.0) | 219 (134, 403) | 2267 (1331, 4219) | <0.001 | <0.001 |
| uAPR (%) | 30.0 (10.8, 57.5) | 6.67 (4.29, 10.0) | 21.5 (13.8, 31.4) | 46.7 (29.6, 58.1) | 65.0 (56.6, 72.6) | <0.001 | <0.001 |
| uNAP (mg/g) | 186 (80.3, 683) | 66.1 (51.1, 88.1) | 109 (75.3, 201) | 289 (167, 589) | 1271 (715, 2436) | <0.001 | <0.001 |

† P-values are calculated by Kruskal-Wallis test for continuous variables and Chi-square test for categorical variables.

‡ P-values for trend are calculated by Spearman's correlation for continuous variables and by Cochran-Armitage trend test for binary variables.

**Abbreviation:** ACEI, angiotensin-converting-enzyme inhibitors; ARBs, angiotensin receptor blockers; CKD, chronic kidney disease; eGFR, estimated glomerular filtration rate; NSAIDs, nonsteroidal anti-inflammatory drugs; OAD, oral antidiabetic agents; uPCR, urine protein-to-creatinine ratio; uACR, urine albumin-to-creatinine ratio; uAPR, urine albumin-to-protein ratio; uNAP: urine non-albumin proteinuria.

**Table S2**. Baseline demographic and clinical characteristics stratified by quartiles of urine protein-to-creatinine ratio (uPCR).

| **Variable** | **Total (n= 2904)** | **uPCR≦101 (n= 722)** | **101 < uPCR≦310 (n= 728)** | **310 < uPCR≦1608 (n= 728)** | **uPCR>1608 (n= 726)** | **P-value**† | **P for trend**‡ |
| --- | --- | --- | --- | --- | --- | --- | --- |
| Age (year), median (IQR) | 58.6 (45.4, 70.8) | 49.5 (36.6, 60.8) | 59.8 (48.3, 72.2) | 63.3 (52.2, 74.9) | 61.1 (48.2, 72.8) | <0.001 | <0.001 |
| Female, n (%) | 1697 (58.4) | 458 (63.4) | 411 (56.5) | 433 (59.5) | 395 (54.4) | 0.003 | 0.003 |
| Body mass index (kg/m^2^), median (IQR) | 24.1 (21.8, 27.3) | 24.17 (21.5, 26.7) | 24.1 (21.7, 27.1) | 24.0 (21.8, 27.3) | 24.4 (21.9, 27.7) | 0.32 | 0.11 |
| Follow-up duration (year), median (IQR) | 3.32 (1.41, 6.28) | 3.47 (1.85, 6.41) | 4.14 (1.97, 7.15) | 2.97 (1.16, 5.96) | 2.71 (0.91, 5.29) | <0.001 | <0.001 |
| **Baseline comorbidities, n (%)** |  |  |  |  |  |  |  |
| CKD stage 1-2 | 1343 (47.3) | 554 (81.1) | 416 (58.2) | 239 (33.3) | 134 (18.6) | <0.001 | <0.001 |
| CKD stage 3-5 | 1495 (52.7) | 129 (18.9) | 299 (41.8) | 479 (66.7) | 588 (81.4) |  |  |
| Diabetes | 928 (32.1) | 108 (15.0) | 233 (32.2) | 249 (34.3) | 338 (46.6) | <0.001 | <0.001 |
| Hypertension | 1245 (43.0) | 163 (22.7) | 300 (41.5) | 345 (47.5) | 437 (60.2) | <0.001 | <0.001 |
| Cardiovascular disease | 766 (26.5) | 101 (14.0) | 190 (26.3) | 212 (29.2) | 263 (36.3) | <0.001 | <0.001 |
| Cancer before enrollment | 265 (9.13) | 40 (5.50) | 84 (11.5) | 74 (10.2) | 67 (9.20) | <0.001 | 0.04 |
| Cancer after enrollment | 166 (5.72) | 23 (3.20) | 45 (6.20) | 56 (7.70) | 42 (5.80) | 0.003 | 0.02 |
| Cancer (before or after enrollment) | 431 (14.8) | 63 (8.70) | 129 (17.7) | 130 (17.9) | 109 (15) | <0.001 | 0.001 |
| **Baseline medication profiles, n (%)** |  |  |  |  |  |  |  |
| Pentoxifylline | 389 (14.8) | 30 (5.20) | 72 (10.9) | 145 (21.4) | 142 (20.1) | <0.001 | <0.001 |
| Dipyridamole | 91 (3.47) | 8 (1.40) | 19 (2.90) | 31 (4.60) | 33 (4.70) | 0.003 | <0.001 |
| Anti-platelet agents | 683 (26.0) | 101 (17.4) | 173 (26.2) | 201 (29.6) | 208 (29.5) | <0.001 | <0.001 |
| NSAIDs | 721 (27.5) | 165 (28.4) | 186 (28.1) | 192 (28.3) | 178 (25.2) | 0.50 | 0.22 |
| Contrast | 479 (18.2) | 90 (15.5) | 124 (18.8) | 143 (21.1) | 122 (17.3) | 0.07 | 0.32 |
| Antihypertension agents |  |  |  |  |  |  |  |
| ACEI | 465 (17.7) | 43 (7.40) | 114 (17.2) | 108 (15.9) | 200 (28.4) | <0.001 | <0.001 |
| ARBs | 919 (35.0) | 116 (20.0) | 230 (34.8) | 252 (37.1) | 321 (45.5) | <0.001 | <0.001 |
| Diuretics | 1192 (45.4) | 132 (22.7) | 258 (39.0) | 315 (46.4) | 487 (69.1) | <0.001 | <0.001 |
| Antidiabetic agents |  |  |  |  |  |  |  |
| OAD | 724 (27.6) | 96 (16.5) | 202 (30.6) | 183 (27.0) | 243 (34.5) | <0.001 | <0.001 |
| Insulin | 539 (20.5) | 31 (5.34) | 101 (15.3) | 169 (24.9) | 238 (33.8) | <0.001 | <0.001 |
| **Baseline biochemical profiles, median (IQR)** |  |  |  |  |  |  |  |
| eGFR (mL/min/1.73m2) | 56.8 (27.8, 91.8) | 91.6 (67.2, 108) | 66.8 (46.7, 95.0) | 42.2 (22.0, 72.5) | 22.7 (8.82, 49.7) | <0.001 | <0.001 |
| Serum creatinine (mg/dL) | 1.23 (0.86, 2.16) | 0.91 (0.74, 1.15) | 1.07 (0.8, 1.44) | 1.53 (1.01, 2.6) | 2.64 (1.35, 5.41) | <0.001 | <0.001 |
| Blood urea nitrogen (mg/dL) | 20 (13, 36) | 13 (10, 17) | 17 (12, 23) | 24 (15, 40) | 40 (22, 66) | <0.001 | <0.001 |
| Serum uric acid (mg/dL) | 6.5 (5.2, 8.0) | 5.8 (4.8, 7.0) | 6.1 (4.9, 7.4) | 6.8 (5.3, 8.4) | 7.4 (6.2, 8.9) | <0.001 | <0.001 |
| Sodium (mmol/L) | 138 (136, 140) | 139 (137, 140) | 138 (136, 140) | 137 (135, 140) | 138 (135, 140) | <0.001 | <0.001 |
| Potassium (mmol/L) | 4.1 (3.7, 4.5) | 4 (3.8, 4.3) | 4 (3.8, 4.4) | 4.1 (3.6, 4.5) | 4.2 (3.8, 4.7) | <0.001 | <0.001 |
| Calcium (mg/dL) | 8.7 (8.2, 9.2) | 9 (8.75, 9.4) | 9 (8.5, 9.3) | 8.8 (8.3, 9.2) | 8.35 (7.8, 8.8) | <0.001 | <0.001 |
| Phosphate (mg/dL) | 4.0 (3.4, 4.8) | 3.7 (3.2, 4.1) | 3.6 (3.1, 4.1) | 3.9 (3.3, 4.6) | 4.5 (3.7, 5.6) | <0.001 | <0.001 |
| Serum albumin (g/dL) | 4.0 (3.3, 4.4) | 4.4 (4.1, 4.6) | 4.2 (3.8, 4.5) | 4 (3.5, 4.3) | 3.3 (2.6, 3.8) | <0.001 | <0.001 |
| Hemoglobin (g/dL) | 11.9 (9.80, 13.8) | 13.6 (12.1, 14.9) | 12.9 (11.1, 14.2) | 11.45 (9.85, 13.3) | 10.1 (8.8, 12.1) | <0.001 | <0.001 |
| Total cholesterol (mg/dL) | 181 (152, 214) | 183.5 (157.5, 213) | 177 (152, 206) | 180 (149, 209) | 188 (153, 231) | <0.001 | <0.001 |
| Triglyceride (mg/dL) | 129 (85, 195) | 116 (76, 173) | 119 (82, 182) | 130.5 (92, 200) | 144 (97, 221) | <0.001 | <0.001 |
| Glucose (mg/dL) | 116 (99, 159) | 106 (96, 131) | 116 (99, 156) | 121 (99, 166) | 126 (101, 176) | <0.001 | <0.001 |
| Urine creatinine (mg/dL) | 92.3 (59.4, 144) | 144 (105, 191) | 96.6 (63.8, 143) | 80.6 (54.7, 119) | 64.8 (46.2, 93.8) | <0.001 | <0.001 |
| uPCR (mg/g) | 310 (101, 1608) | 68.5 (54.7, 84.5) | 160 (126, 218) | 672 (440, 1033) | 3848 (2404, 6827) | <0.001 | <0.001 |
| uACR (mg/g) | 86.1 (12.8, 757) | 5.70 (3.37, 11.5) | 36.7 (15.8, 68.3) | 249 (123, 511) | 2267 (1331, 4219) | <0.001 | <0.001 |
| uAPR (%) | 30.0 (10.8, 57.5) | 8.89 (5.56, 16.7) | 22.9 (10.3, 39.2) | 45.2 (20.5, 61.3) | 62.2 (51.3, 70.8) | <0.001 | <0.001 |
| uNAP (mg/g) | 186 (80.3, 683) | 58.9 (47.7, 71.6) | 114 (94.2, 154) | 347 (236, 529) | 1575 (933, 2670) | <0.001 | <0.001 |

† P-values are calculated by Kruskal-Wallis test for continuous variables and Chi-square test for categorical variables.

‡ P-values for trend are calculated by Spearman's correlation for continuous variables and by Cochran-Armitage trend test for binary variables.

**Abbreviation:** ACEI, angiotensin-converting-enzyme inhibitors; ARBs, angiotensin receptor blockers; CKD, chronic kidney disease; eGFR, estimated glomerular filtration rate; NSAIDs, nonsteroidal anti-inflammatory drugs; OAD, oral antidiabetic agents; uPCR, urine protein-to-creatinine ratio; uACR, urine albumin-to-creatinine ratio; uAPR, urine albumin-to-protein ratio; uNAP: urine non-albumin proteinuria.

**Table S3.** Hazard Ratios (95% confidence interval) of risk of all-cause mortality by uPCR, uACR, uAPR, and uNAP by the concordance between uPCR and uACR and uAPR above or below 40% (based on imputation dataset and model with time-on-study as the time-scale).

|  | **Crude model (95% CI)** | **Model 1 (95% CI)** | **Model 2 (95% CI)** | **Model 3 (95% CI)** |
| --- | --- | --- | --- | --- |
| **Overall** |  |  |  |  |
| uPCR (per double increase) | **1.35 (1.31, 1.39)** | **1.39 (1.34, 1.44)** | **1.39 (1.34, 1.44)** | **1.27 (1.22, 1.33)** |
| uACR (per double increase) | **1.19 (1.16, 1.22)** | **1.20 (1.17, 1.23)** | **1.20 (1.17, 1.24)** | **1.11 (1.08, 1.15)** |
| uAPR (per 10% increase) | **1.07 (1.04, 1.10)** | **1.07 (1.03, 1.10)** | **1.07 (1.03, 1.10)** | 1.01 (0.98, 1.05) |
| uNAP (per double increase) | **1.48 (1.43, 1.54)** | **1.51 (1.45, 1.58)** | **1.52 (1.46, 1.58)** | **1.38 (1.31, 1.45)** |
| **Concordance proteinuria** |  |  |  |  |
| uPCR (per double increase) | **1.36 (1.31, 1.41)** | **1.40 (1.35, 1.46)** | **1.41 (1.35, 1.47)** | **1.28 (1.22, 1.35)** |
| uACR (per double increase) | **1.24 (1.20, 1.27)** | **1.26 (1.22, 1.30)** | **1.26 (1.22, 1.30)** | **1.18 (1.13, 1.22)** |
| uAPR (per 10% increase) | **1.17 (1.13, 1.21)** | **1.17 (1.13, 1.22)** | **1.17 (1.13, 1.22)** | **1.10 (1.06, 1.15)** |
| uNAP (per double increase) | **1.48 (1.42, 1.54)** | **1.51 (1.44, 1.58)** | **1.51 (1.44, 1.58)** | **1.36 (1.28, 1.44)** |
| **Non-albumin predominant proteinuria** |  |  |  |  |
| uPCR (per double increase) | **1.46 (1.30, 1.63)** | **1.49 (1.31, 1.69)** | **1.49 (1.31, 1.70)** | **1.39 (1.19, 1.62)** |
| uACR (per double increase) | **1.15 (1.05, 1.25)** | **1.14 (1.04, 1.25)** | **1.14 (1.04, 1.25)** | 1.07 (0.96, 1.18) |
| uAPR (per 10% increase) | 0.87 (0.75, 1.01) | 0.87 (0.75, 1.02) | 0.88 (0.75, 1.03) | 0.86 (0.73, 1.01) |
| uNAP (per double increase) | **1.46 (1.31, 1.62)** | **1.50 (1.32, 1.69)** | **1.50 (1.33, 1.70)** | **1.41 (1.21, 1.64)** |
| **uAPR < 40%** |  |  |  |  |
| uPCR (per double increase) | **1.71 (1.62, 1.79)** | **1.65 (1.56, 1.75)** | **1.65 (1.56, 1.75)** | **1.54 (1.43, 1.67)** |
| uACR (per double increase) | **1.47 (1.40, 1.54)** | **1.37 (1.30, 1.45)** | **1.38 (1.31, 1.45)** | **1.23 (1.15, 1.31)** |
| uNAP (per double increase) | **1.72 (1.64, 1.81)** | **1.67 (1.58, 1.78)** | **1.67 (1.58, 1.78)** | **1.57 (1.45, 1.70)** |
| **uAPR ≥ 40%** |  |  |  |  |
| uPCR (per double increase) | **1.30 (1.23, 1.38)** | **1.39 (1.30, 1.48)** | **1.39 (1.31, 1.49)** | **1.25 (1.16, 1.35)** |
| uACR (per double increase) | **1.25 (1.18, 1.32)** | **1.34 (1.26, 1.42)** | **1.35 (1.27, 1.43)** | **1.22 (1.14, 1.31)** |
| uNAP (per double increase) | **1.36 (1.28, 1.45)** | **1.42 (1.33, 1.51)** | **1.42 (1.34, 1.52)** | **1.27 (1.17, 1.37)** |

Model 1: Adjusted gender, diabetes, hypertension, cardiovascular disease and cancer.

Model 2: Adjusted gender, diabetes, hypertension, cardiovascular disease, cancer, ACEI, ARBs, and anti-platelet agents.

Model 3: Adjusted gender, diabetes, hypertension, cardiovascular disease, cancer, ACEI, ARBs, anti-platelet agents, eGFR, hemoglobin, and glucose.

**Abbreviation:** ACEI, angiotensin-converting-enzyme inhibitors; ARBs, angiotensin receptor blockers; eGFR, estimated glomerular filtration rate; uAPR, urine albumin-to-protein ratio.

**Table S4**. Hazard ratios (HRs) and 95% CIs of uPCR and uACR for all-cause mortality by uAPR at different cut-off values from 30% to 70%. Adjustment factors were the same as those for Model 3 in Table 4 (based on imputation dataset).

|  |  |  | **uPCR  (mg/g, per double increase)** | |  | **uACR  (mg/g, per double increase)** | |  | **uNAP (mg/g, per double increase)** | |
| --- | --- | --- | --- | --- | --- | --- | --- | --- | --- | --- |
|  | **N** | **Case** | **Adjusted HR  (95% CI)** | **P for  interaction** |  | **Adjusted HR  (95% CI)** | **P for  interaction** |  | **Adjusted HR  (95% CI)** | **P for  interaction** |
| uAPR (%) |  |  |  |  |  |  |  |  |  |  |
| <30% | 1446 | 265 | 1.67 (1.52, 1.84) | **0.001** |  | 1.33 (1.23, 1.43) | 0.286 |  | 1.68 (1.53, 1.85) | **0.002** |
| ≧30% | 1458 | 392 | 1.29 (1.21, 1.38) |  |  | 1.24 (1.17, 1.31) |  |  | 1.32 (1.23, 1.42) |  |
| <40% | 1683 | 320 | 1.64 (1.51, 1.79) | **0.001** |  | 1.25 (1.17, 1.34) | 0.889 |  | 1.67 (1.54, 1.82) | **<0.001** |
| ≧40% | 1221 | 337 | 1.27 (1.18, 1.37) |  |  | 1.24 (1.16, 1.33) |  |  | 1.28 (1.19, 1.39) |  |
| <50% | 1931 | 395 | 1.49 (1.39, 1.59) | 0.318 |  | 1.19 (1.13, 1.25) | 0.159 |  | 1.56 (1.45, 1.68) | 0.083 |
| ≧50% | 973 | 262 | 1.33 (1.21, 1.46) |  |  | 1.30 (1.19, 1.43) |  |  | 1.31 (1.20, 1.44) |  |
| <60% | 2255 | 505 | 1.37 (1.30, 1.46) | 0.163 |  | 1.14 (1.10, 1.19) | **0.003** |  | 1.48 (1.38, 1.57) | 0.777 |
| ≧60% | 649 | 152 | 1.41 (1.23, 1.61) |  |  | 1.41 (1.23, 1.61) |  |  | 1.34 (1.19, 1.52) |  |
| <70% | 2607 | 608 | 1.34 (1.28, 1.40) | 0.961 |  | 1.14 (1.10, 1.18) | 0.271 |  | 1.45 (1.38, 1.53) | 0.112 |
| ≧70% | 297 | 49 | 1.25 (0.96, 1.62) |  |  | 1.30 (0.99, 1.70) |  |  | 1.10 (0.88, 1.37) |  |

**Table S5**. The international classification of disease of comorbidities.

| **Category** | **Name** | **ICD codes** |
| --- | --- | --- |
| Comorbidity | Cardiovascular disease | **ICD-9-CM:**  410-414, 425-428, 441-442, 458, 250.7, 429.1-429.3, 430-438, 443.9, 785.4, V43.4 |
| Comorbidity | Diabetes | **ICD-9-CM:**  250 |
| Comorbidity | Hypertension | **ICD-9-CM:**  401-405 |
| Comorbidity | Cancer | **ICD-9-CM:**  140-208  **ICD-10-CM:**  C00-C97 |

ICD-9-CM, International Classification of Diseases, 9th Revision, Clinical Modification

ICD-10-CM, International Classification of Diseases, 10th Revision, Clinical Modification

**Table S6**. The number of patients with available information for the baseline demographic and clinical characteristics in the study population (n = 2904 patients).

|  | | **Number of patients with available information** | |
| --- | --- | --- | --- |
| Age | | 2904 | |
| Female | | 2904 | |
| Body mass index (kg/m^2^) | | 1394 | |
| Follow-up duration (year) | | 2904 | |
| **Baseline comorbidities** | |  | |
| CKD stage | | 2838 | |
| Diabetes | | 2894 | |
| Hypertension | | 2894 | |
| Cardiovascular disease | | 2892 | |
| Cancer before enrollment | | 2904 | |
| Cancer after enrollment | | 2904 | |
| Cancer (before or after enrollment) | | 2904 | |
| **Baseline medication profiles** | |  | |
| Pentoxifylline | | 2626 | |
| Dipyridamole | | 2626 | |
| Anti-platelet agents | | 2626 | |
| NSAIDs | | 2626 | |
| Contrast | | 2626 | |
| ACEI | | 2626 | |
| ARBs | | 2626 | |
| Diuretics | | 2626 | |
| OAD | | 2626 | |
| Insulin | | 2626 | |
| **Baseline biochemical profiles** | |  | |
| Serum creatinine (mg/dL) | | 2838 | |
| Blood urea nitrogen (mg/dL) | | 2612 | |
| Serum uric acid (mg/dL) | | 2398 | |
| Sodium (mmol/L) | | 2159 | |
| Potassium (mmol/L) | | 2364 | |
| Calcium (mg/dL) | | 1541 | |
| Phosphate (mg/dL) | | 1280 | |
| Serum albumin (g/dL) | | 2209 | |
| Hemoglobin (g/dL) | | 2059 | |
| Total cholesterol (mg/dL) | | 2021 | |
| Triglyceride (mg/dL) | | 2087 | |
| Glucose (mg/dL) | | 2446 | |
| Urine creatinine (mg/dL) | | 2904 | |
| uPCR (mg/g) | | 2904 | |
| uACR (mg/g) | | 2904 | |
| uAPR (%) | | 2904 | |
| uNAP (mg/g) | | 2904 | |

**Figure S1.** The classification matrix base on severity grades of both uACR and uPCR and categorized patients into three group (concordance proteinuria, non-albumin predominant proteinuria, and albumin predominant proteinuria).


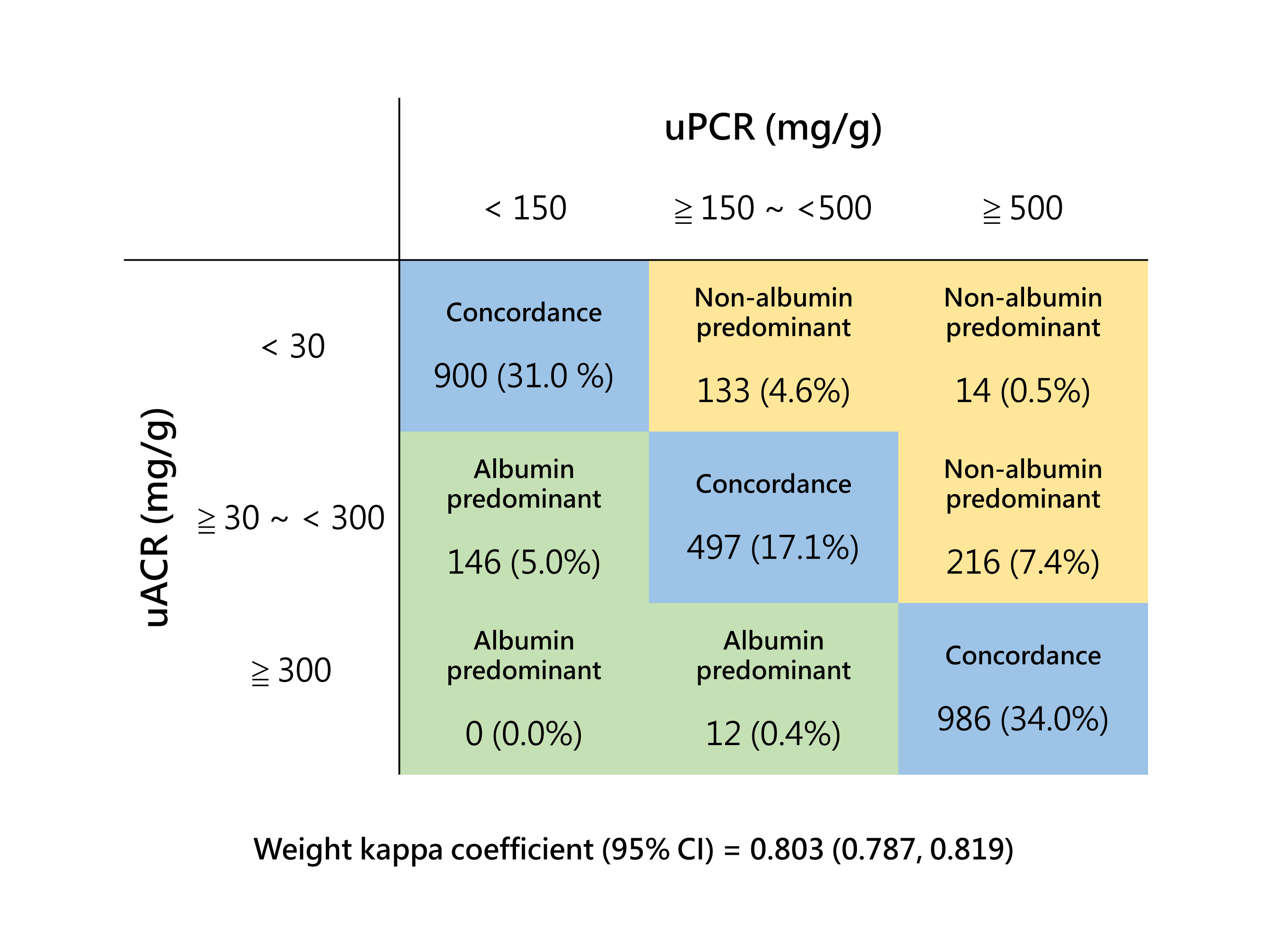


**Figure S2.** Hazard ratios (HRs) for all-cause mortality according to uPCR, uACR, uAPR, and uNAP in each concordant proteinuria. Solid lines represent adjusted HRs based on restricted cubic splines for each urinary biomarker with knots at the 10th, 50th, and 90th percentiles. Shaded areas represent 95% confidence interval. The reference was set at the 10th percentile of each urinary biomarker. The blue, green and dark-red lines and shaded areas represent the concordant proteinuria from normal: uPCR <150 mg/g creatinine and uACR <30 mg/g creatinine; moderate: 150≦uPCR <500 mg/g creatinine and 30≦uACR <300 mg/g creatinine; severe: creatinine to uPCR ≧500 mg/g creatinine and uACR ≧300 mg/g creatinine, respectively (based on imputation dataset).


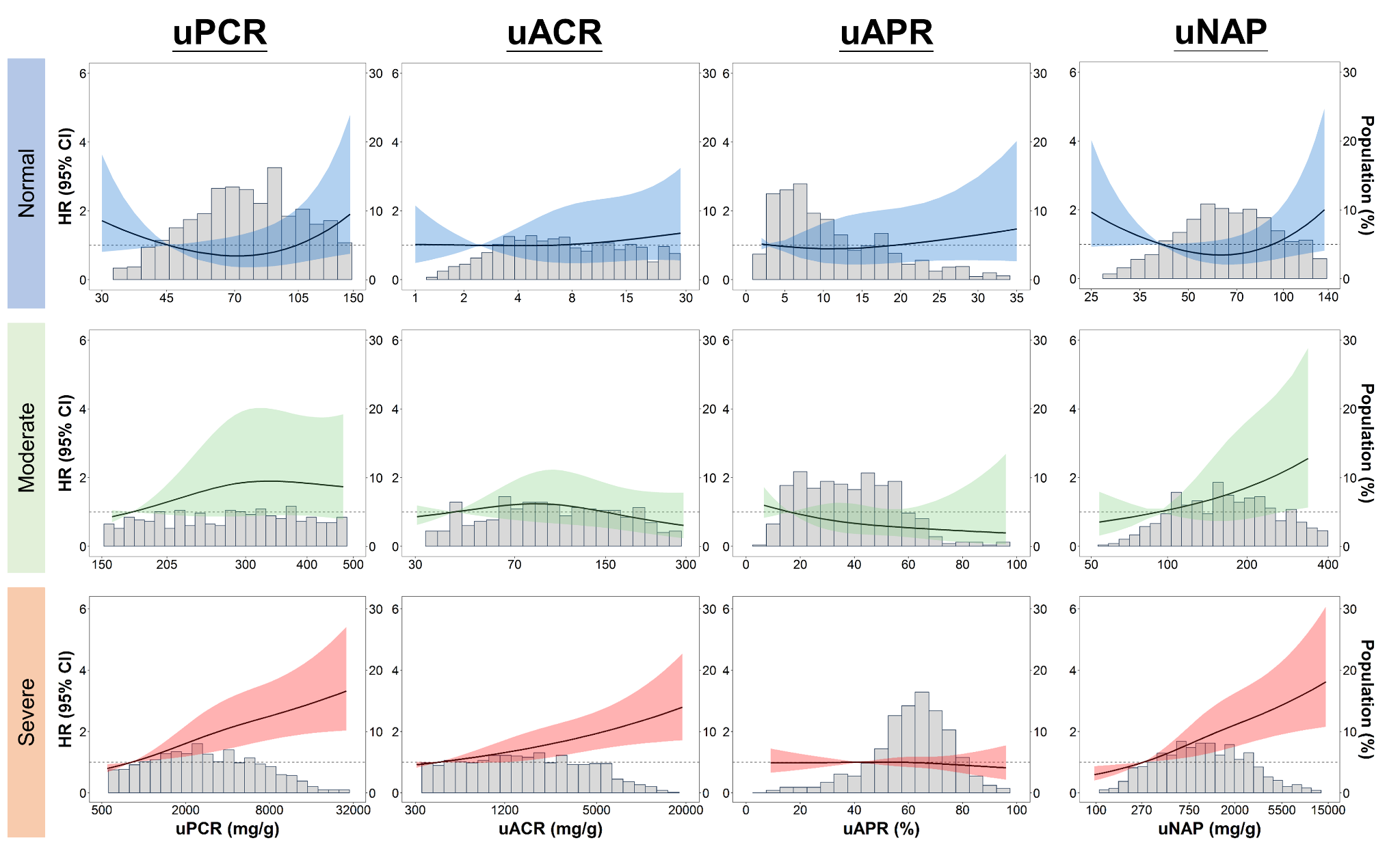


**Figure S3.** Hazard ratios (HRs) and 95% CIs for the risk of all-cause mortality comparing the moderate (150≦uPCR <500 mg/g creatinine and 30≦uACR <300 mg/g creatinine) and server (creatinine to uPCR ≧500 mg/g creatinine and uACR ≧300 mg/g creatinine) verse normal (uPCR <150 mg/g creatinine and uACR <30 mg/g creatinine) in concordant proteinuria, by patient characteristics at baseline. Adjustment factors were the same as those for Model 3 in Table 4 (based on imputation dataset).

**
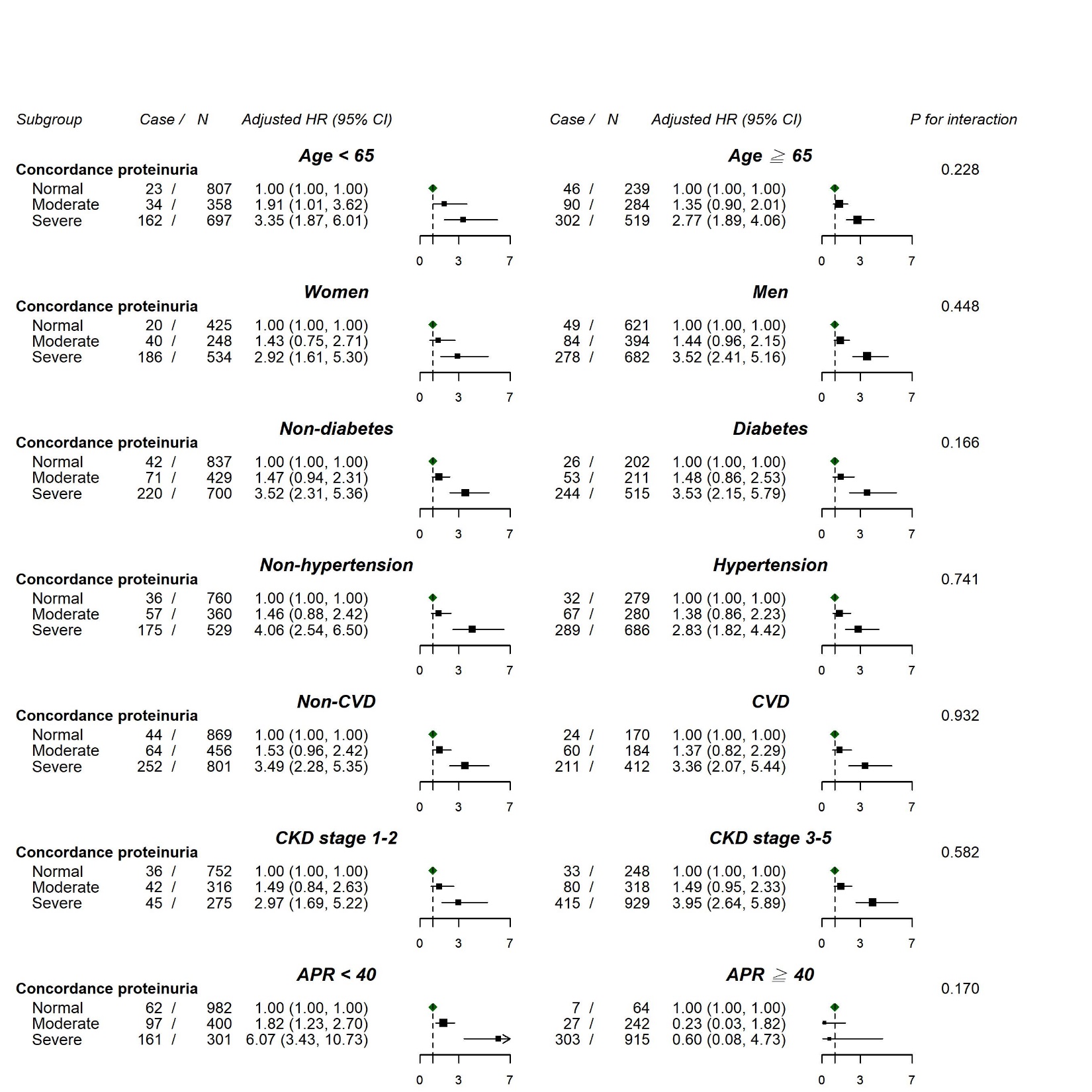
**

**Figure S4.** Discrimination statistics (based on an imputed database) and calibration plots (based on an actual database with the outcome of 3-year all-cause mortality) for reference (Model 3 in Table 4) and new models (Model 3 + uNAP, Model 3 + uPCR, Model 3 + uACR, and Model 3 + uAPR).


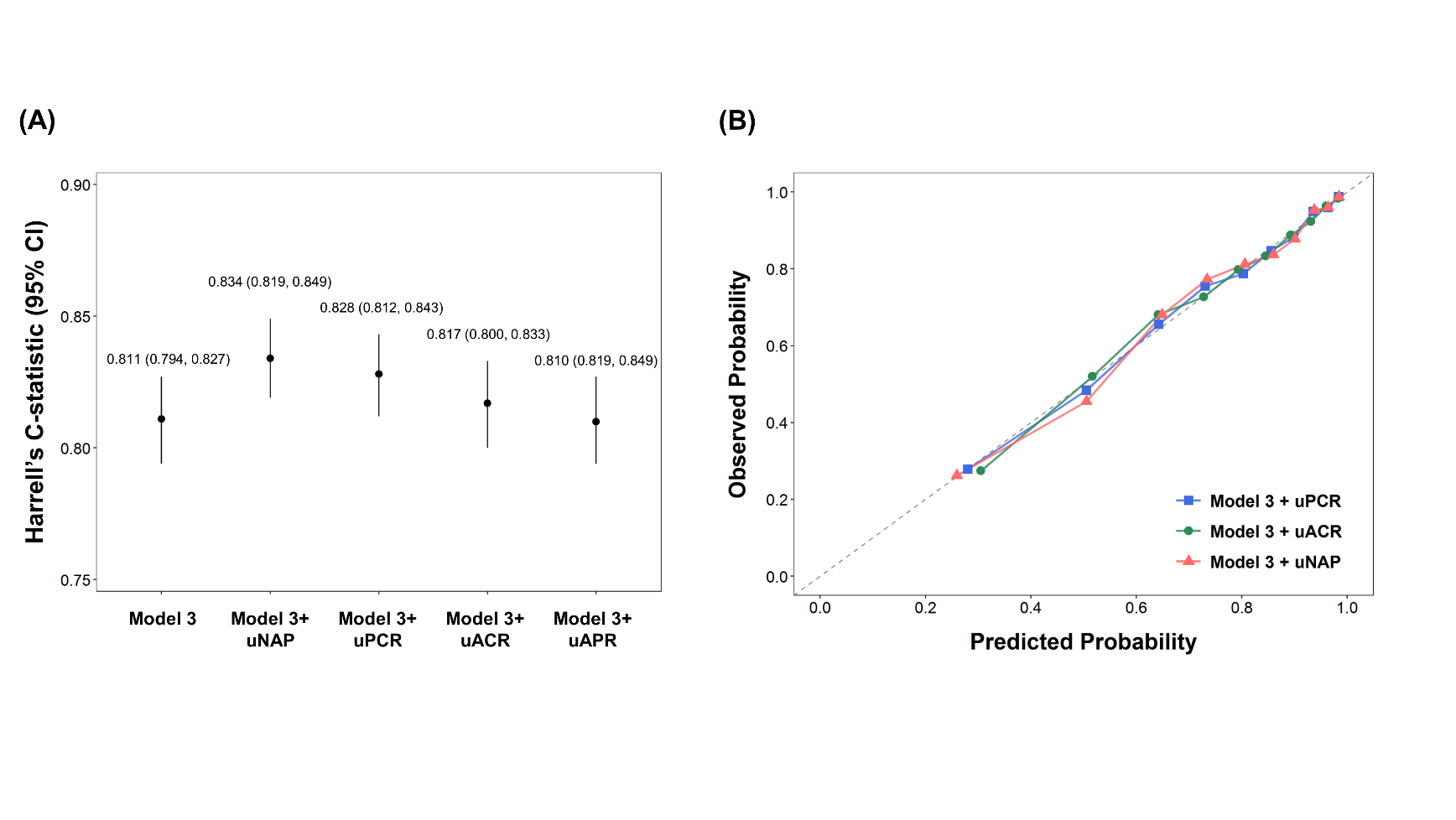


|  | **Harrell’s c statistic**  **(95% CI)** | **P-value** |
| --- | --- | --- |
| Model 3 | 0.811  (0.794, 0.827) | - |
| Model 3 + uNAP (mg/g) | 0.834  (0.819, 0.849) | Ref |
| Model 3 + uPCR (mg/g) | 0.828  (0.812, 0.843) | 0.032 |
| Model3 + uACR (mg/g) | 0.817  (0.800, 0.833) | 0.004 |
| Model3 + uAPR (%) | 0.810  (0.794, 0.827) | <0.001 |

**Figure S5a.** Cut-off determination of uPCR, uACR, uAPR, and uNAP for all-cause mortality by patients’ proteinuric classifications**.** uPCR, urine protein-to-creatinine ratio; uACR, urine albumin-to-creatinine ratio; uAPR, urine albumin-to-protein ratio; uNAP, urine non-albumin proteinuria.


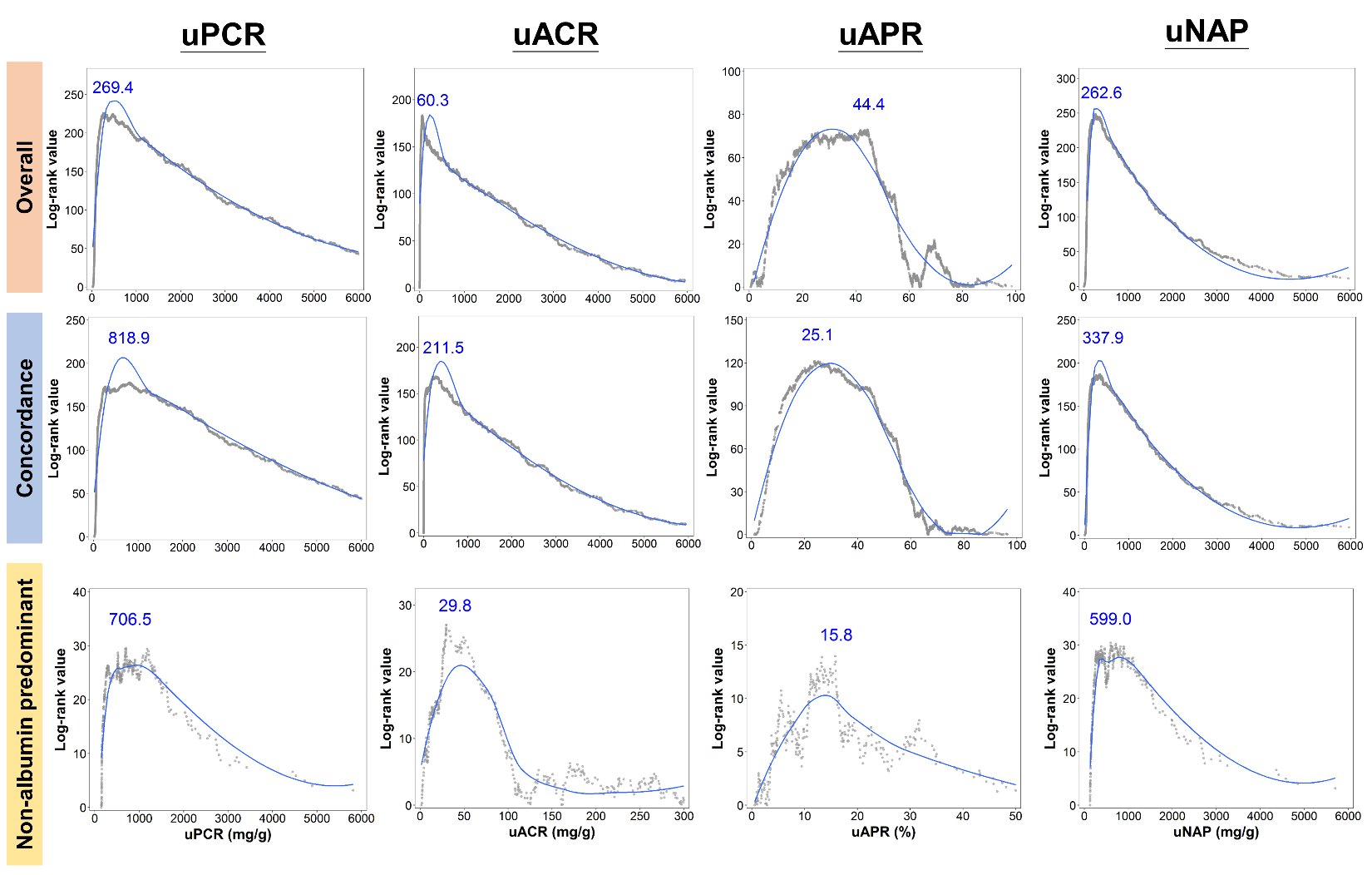


**Figure S5b.** Cut-off determination of uPCR, uACR, uAPR, and uNAP for all-cause mortality by age cutoff of 65 years**.** uPCR, urine protein-to-creatinine ratio; uACR, urine albumin-to-creatinine ratio; uAPR, urine albumin-to-protein ratio; uNAP, urine non-albumin proteinuria.


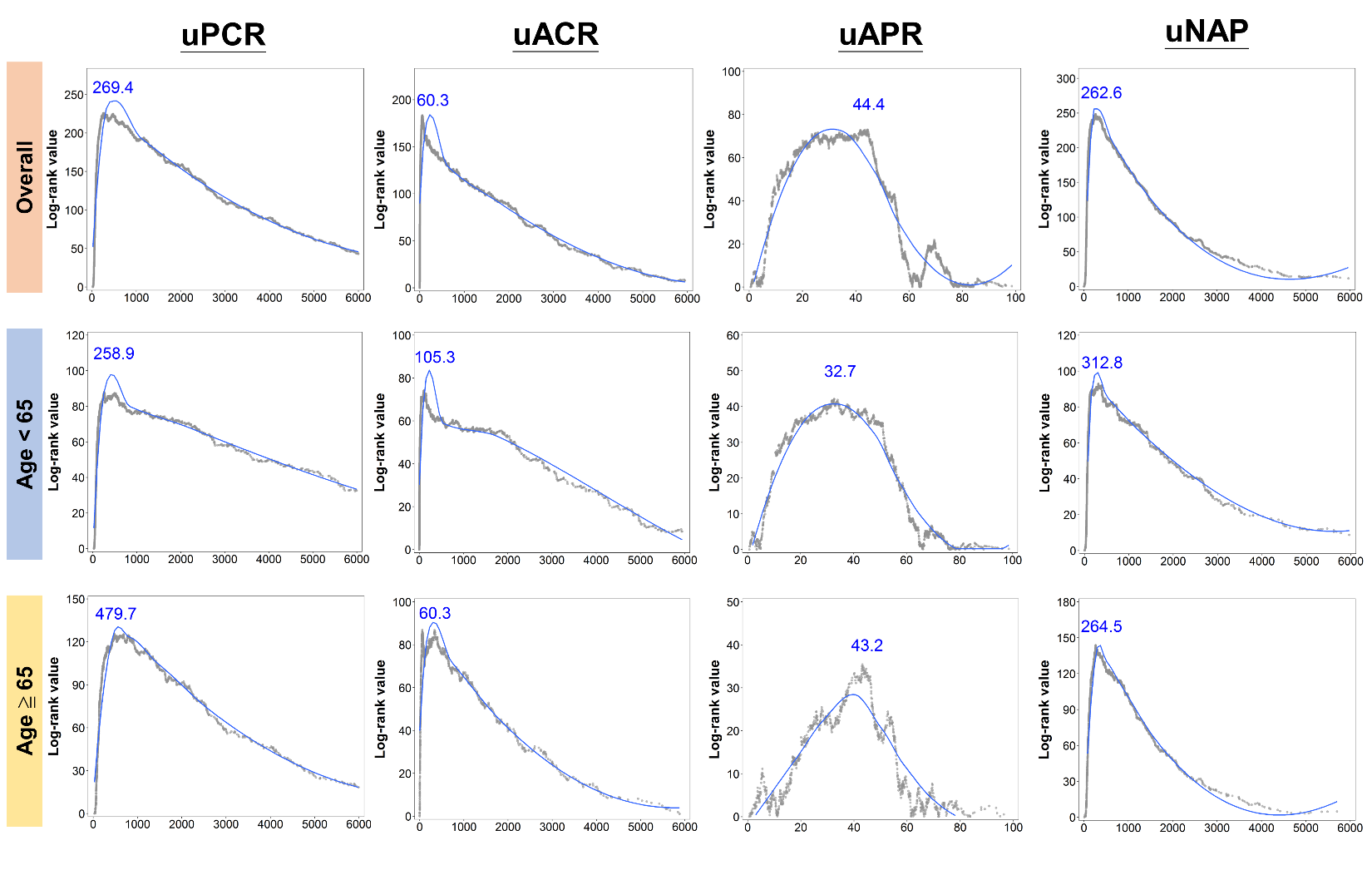


**Figure S5c.** Cut-off determination of uPCR, uACR, uAPR, and uNAP for all-cause mortality by patients’ sex**.** uPCR, urine protein-to-creatinine ratio; uACR, urine albumin-to-creatinine ratio; uAPR, urine albumin-to-protein ratio; uNAP, urine non-albumin proteinuria.


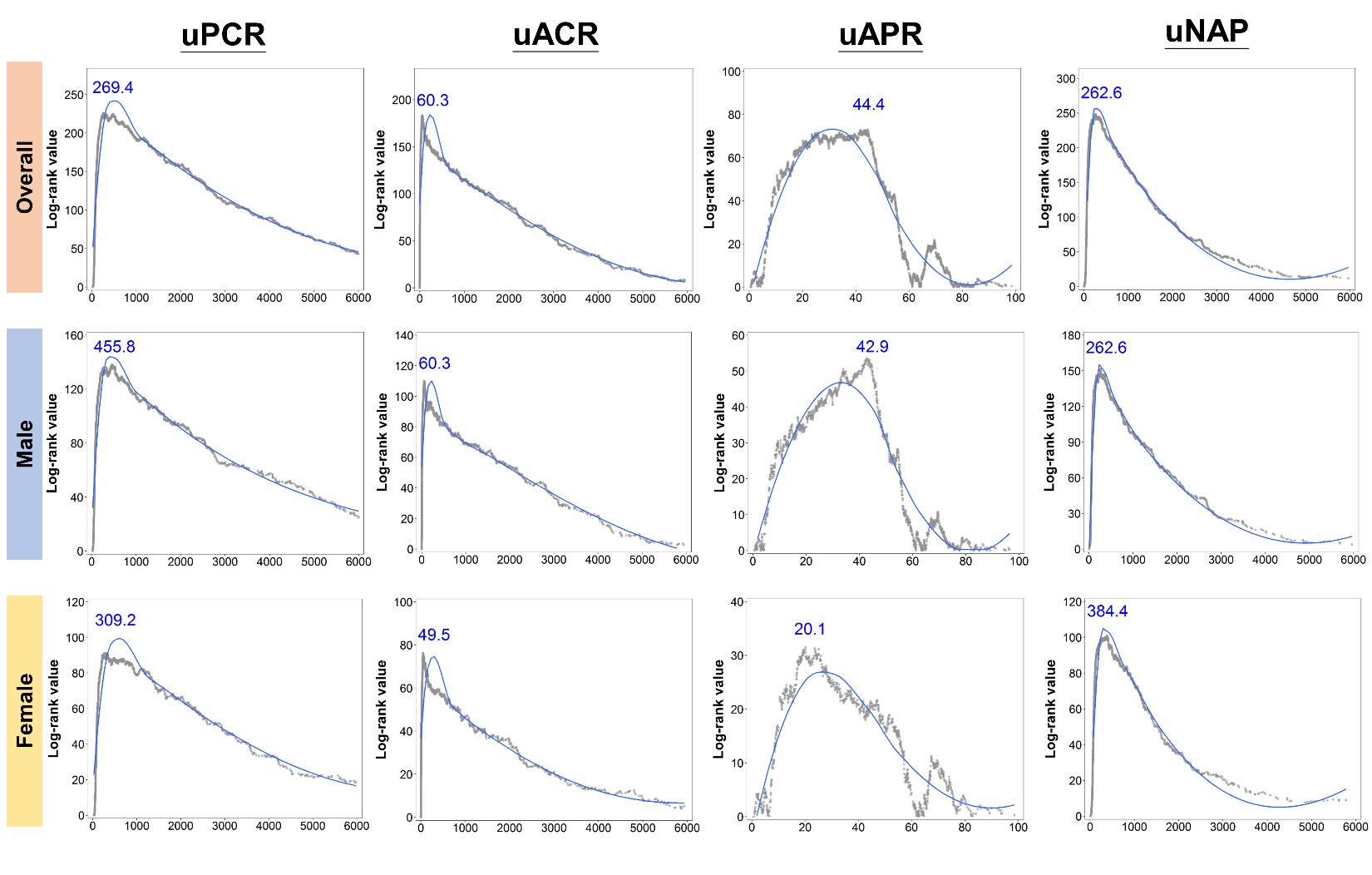


**Figure S6.** Flow diagram of patient selection.


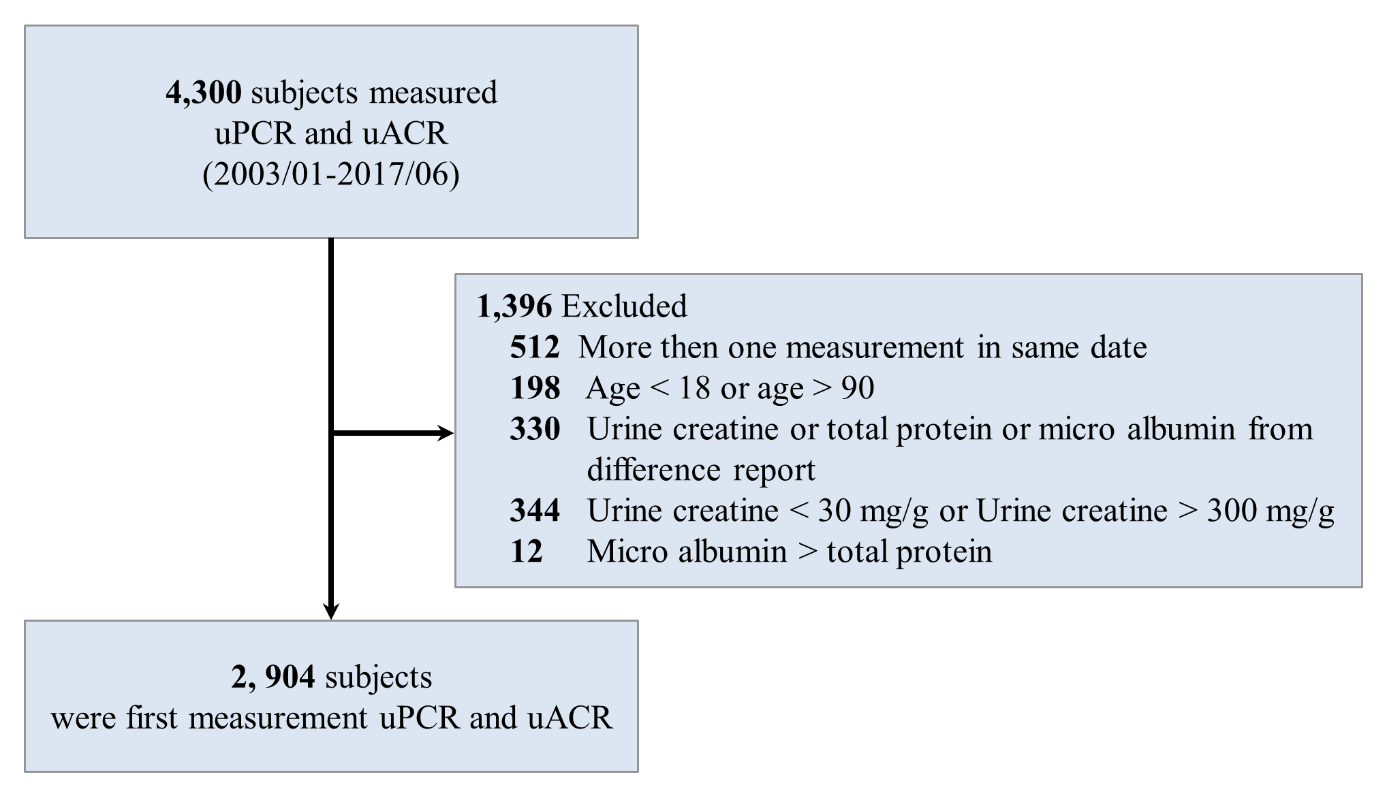

Supplement: Supplementary file 1 — Supplementary Information. [file 41598_2021_86541_MOESM1_ESM.docx]
